# Supplementary material for: Short-term effects of GPS collars on the activity, behavior, and adrenal response of scimitar-horned oryx (Oryx dammah)
Source: PLoS One. 2020 Feb 11;15(2):e0221843. doi: 10.1371/journal.pone.0221843 (PMC7012457; doi:10.1371/journal.pone.0221843)
Supplement: S1 Code — Script and data tutorial to investigate behavioral changes observed in scimitar-horned oryx (Oryx dammah) fit with GPS collars. Data fit in a Bayesian framework, estimating the probability of each behavioral activity and based on a multinomial likelihood. Each animal was used as their own control to assess how each behavior changed across time periods (Pre-treatment, Treatment, Post-treatment). (ZIP) [file pone.0221843.s001.zip › S1_Code/BehaviorComparison_MultinomialRegression.html]

Multinomial Logistic Regression: Observation Data


# Multinomial Logistic Regression: Observation Data

### *Stabach et al. 2019 - Effects of GPS Collars*

#### *Jared Stabach, Smithsonian Conservation Biology Institute*

#### *2019-December-18*

# Multinomial Regression

> Comments/Questions: Contact Grant Connette (connetteg@si.edu) and Jared Stabach (stabachj@si.edu)

Data analysis to investigate behavioral changes observed in scimitar-horned oryx (*Oryx dammah*) fitted with GPS collars and described in Stabach et al. 2019. Data fit in a Bayesian framework, estimating the probability of different behaviors using a multinomial logistic regression model. Each animal was used as their own control to assess how each behavior changed across time periods (Pre-treatment, Treatment, Post-treatment). Our expectation was that potential adverse behaviors, such as headshaking, should increase during the period immediately after animals were collared (Treatment Period) and return to normal activity during the Post-treatment period when animals acclimate or adjust to the device.

The steps described here are aimed at recreating the figures and tables listed in the manuscript.

Additional details in:

Stabach, J.A., S.A. Cunningham, G. Connette, J.L. Mota, D. Reed, M. Byron, M. Songer, T. Wacher, K. Mertes, J.L. Brown, P. Comizzoli, J. Newby, S. Monfort, and P. Leimgruber. In Review. Short-term effects of GPS collars on behavior and stress of scimitar-horned Oryx (*Oryx dammah*). PlosOne.

### Load Libraries

Load each library necessary to complete the analysis.

```
# Clear objects in memory
rm(list=ls())

# Load necessary libraries
library(tidyr)
library(reshape2)
library(ggplot2)
library(jagsUI)
library(MCMCvis)
library(HDInterval)
```

### Read/Prepare Data

Import the dataframe into R and correct the datatypes for some of the variables (`as.POSIXct()` and `as.factor()`). Remove the ‘Control’ animals from the dataframe since the number of individuals was too low to use in analyses. Collared animals served as their own control, since pre-treatment information was collected.

```
# Read in file
bdata <- read.csv("./Data/bdata.csv", header=T, sep=",", row.names=1)

# View data
head(bdata)
```

```
##        Date           TimeStart             TimeEnd Elapsed Temperature
## 1 10/8/2015 2015-10-08 10:23:00 2015-10-08 10:33:00      10          19
## 2 10/8/2015 2015-10-08 10:23:00 2015-10-08 10:33:00      10          19
## 3 10/8/2015 2015-10-08 10:23:00 2015-10-08 10:33:00      10          19
## 4 10/8/2015 2015-10-08 10:23:00 2015-10-08 10:33:00      10          19
## 5 10/9/2015 2015-10-09 08:45:00 2015-10-09 08:55:00      10          18
## 6 10/9/2015 2015-10-09 08:45:00 2015-10-09 08:55:00      10          18
##   Weather Location Barn.in.out.  Animal    Sex Treatment Collard
## 1   Sunny    Meade           in Loretta Female   control      No
## 2   Sunny    Meade           in    Ruby Female       ATS      No
## 3   Sunny    Meade           in   Scout Female       ATS      No
## 4   Sunny    Meade           in  Bamako Female       ATS      No
## 5   Sunny    Meade           in  Violet Female Vectronic      No
## 6   Sunny    Meade           in  Bamako Female       ATS      No
##   CollarIssue RelDay AdjObTime TotalObs ModTotObs HU HD LAY HDSK LOCO
## 1          No    -14         1       40        40 38  0   0    0    2
## 2          No    -14         1       40        40 30  1   0    6    1
## 3          No    -14         1       39        40 29  1   0    0    6
## 4          No    -14         1       36        40 29  1   0    0    4
## 5          No    -13         1       36        40  9  0  18    8    1
## 6          No    -13         1       40        40  8  0  30    0    2
##   SCRATCH OOV RSums
## 1       0   0     1
## 2       2   0     1
## 3       3   1     1
## 4       2   4     1
## 5       0   4     1
## 6       0   0     1
```

```
# Set/Update the data/time fields
bdata$TimeStart <- as.POSIXct(bdata$TimeStart, format="%Y-%m-%d %H:%M")
bdata$TimeEnd <- as.POSIXct(bdata$TimeEnd, format="%Y-%m-%d %H:%M")

# Code the Control and Treatment records
# Remove the Controls, too few animals to be useful
# Code the Control and Treatment records.
bdata$Control <- ifelse(bdata$Treatment == "control",1,2) 
bdata.control <- bdata[which(bdata$Treatment == "control"),]
bdata <- bdata[which(bdata$Treatment != "control"),]

# Set AdjObTime as a factor 
bdata$AdjObTime <- as.factor(bdata$AdjObTime)
```

### Format for JAGS

To prepare the data to be ingested into **JAGS**, we first set the number of iterations, the burn-in rate (20%), and the thinning rate of our Markov chain. Note, the number of iterations (`n.iter`) has been reduced in the code (i.e., set to 100,000) so that analyses will execute quickly. `n.iter` should be increased in subsequent analyses to make sure the parameter space has been thoroughly explored.

For results presented in our manuscript, we ran three parallel Markov chain Monte Carlo (MCMC) chains for 500,000 iterations, discarding the first 100,000 iterations as burn-in, and thinned the remaining posterior samples at a rate of 1:100. The joint posterior distribution, therefore, yielded a total of 12,000 samples. Convergence was assessed by visual inspection of traceplots to ensure a reasonable exploration of the parameter space and by ensuring that the potential scale reduction factor was < 1.1 for each parameter (Gelman & Rubin 1992).

```
# Set-up burn-in/iterations for JAGS
n.iter=100000 # Number of iterations.  
n.burnin=n.iter*0.20 # burn-in iterations (0.20 percent)
n.thin = 100

# Set up blank list
data.list <- vector("list")

y <- cbind(bdata$HU,bdata$HD,bdata$LAY,bdata$HDSK,bdata$LOCO,bdata$SCRATCH) 
#class(y) To make sure apended to matrix

# Create matrix for inverse Wishart prior on individual random effects
R <- matrix(0,nrow=6,ncol=6)
for (i in 1:6){
  R[i,i] <- 0.1
}

# Setup the data list
data.list=list(
  Y = y, 
  n.outcomes = ncol(y),
  PERIOD = as.numeric(bdata$AdjObTime),
  N = apply(y,1,sum),
  n = nrow(y),
  ind = as.numeric(droplevels(bdata$Animal)),
  nind = length(unique(bdata$Animal)),
  R = R
)
```

### Model Description

To assess differences in the frequencies of observed response behaviors between treatment periods, we fit a multinomial logistic regression model to the data. The behavioral observations for each 10-minute observation period were represented as a vector of counts, \(C\_{i.} = (C\_{i1},\dots,C\_{i6})\), summarizing the number of times each of the six response behaviors were observed during a given observation period, \(i\). Each vector of behavioral counts was treated as a multinomial response:

\[ C\_{i.}\sim Multinomial(p\_{i.},N\_i)\]

where \(p\_{i.}=(p\_{i1},\dots,p\_{i6})\) represented the probability of each response behavior during observation period \(i\_.\) and \(N\_i\) was an integer value indicating the total number of behavioral observations during the observation period (\(N\_i\) = 10 minutes/period \* 4 observations/minute = 40 observations/period).

We modeled response probabilities, \(p\_{ij}\), for a given observation period, \(i\), as a function of both individual and treatment period, where the log odds of each possible response behavior, \(j\), are modelled as a linear combination of predictor variables:

\[p\_{ij} = \frac{\varphi\_{ij}}{\Sigma\_j\varphi\_{ij}} \] \[log(\varphi\_{ij}) = \alpha\_j + \beta\_{1,j}Period1\_i+\beta\_{2,j}Period2\_i+\gamma\_{Ind(i)j} \] where \(\alpha\_j\) represented the baseline (Pre-treatment) regression intercept for response behavior \(j\), \(\beta\_{1,j}\) represented the corresponding regression coefficient associated with the treatment period, and \(\beta\_{2,j}\) represented the regression coefficient associated with the post-treatment period. The binary indicator variables, \(Period1\_i\) and \(Period2\_i\), designated whether or not each observation period, \(i\), occurred during either the Treatment (\(Period1\)) or Post-treatment period (\(Period2\)). An individual random error term, \(\gamma\_{Ind(i)j}\), was incorporated to control for the effects of repeated measurements of the same individual and uneven numbers of observation periods for each individual among treatment periods. Individual errors, \(\gamma\_{Ind(i).}\), were assumed to be realizations of a multivariate normal distribution with an all-zero mean vector, \(\mu\_{i.}\), and a variance-covariance matrix, \(\Sigma\), that defined the variation among individuals in the frequencies of each response behavior (diagonal elements) as well as the correlation among behavioral responses within observation periods (off-diagonal elements): \[\gamma\_{Ind(i),}\sim MvNormal(\mu\_i,\Sigma)\] We assigned diffuse \(Normal(\mu = 0, \sigma^2 = 10^3)\) priors for the Pre-treatment intercept terms, \(\alpha\_j\), and the regression coefficients corresponding to the Treatment and Post-treatment periods, \(\beta\_{1,j}\) and \(\beta\_{2,j}\). We assigned a Wishart prior, parameterized by a scale matrix and a degrees of freedom parameter, to the inverse of the variance-covariance matrix, \(\Sigma\). We set the degrees of freedom equal to 7 (\(df = j + 1\)), the diagonal elements of the scale matrix equal to 1, and the off-diagonal elements equal to 0. Finally, for parameter identifiability, the first behavioral response (\(j = 1\); Standing head-Up) was selected as a reference category and the corresponding parameters \(\alpha\_1\), \(\beta\_{1,1}\), and \(\beta\_{2,1}\) were fixed to a value of 0.

The model is saved as `Model_Multinomial_withREs.R` and sourced in the `jags` function below.

```
model{
  
  # SPECIFY THE PRIORS FOR GLOBAL PARAMETERS
  # ***********************************
  # Alphas represent the intercept for relative probabilities of each outcome at the pre-treatment period on the log scale
  # Fix the relative probability of the reference outcome (HU) to zero on the log scale
  alpha[1] <- 0  
  
  # Loop over response outcomes
  for (j in 2:n.outcomes) {   
    # Assign diffuse priors to the relative probabilities of all outcomes except the reference (for period 1)
    alpha[j] ~ dnorm(0, 0.001)  
  }
  
  # Betas represent change from period one on the log scale
  # Loop over response outcomes
  for (j in 1:n.outcomes){
    # beta[1,] are fixed to zero because there is no period-adjustment needed for period 1 since it is the reference
    beta[1, j] <- 0   
  }
  
  # Loop over time periods 2 (treatment) and 3 (post-treatment)
  for (i in 2:3) { 
    # As for period 1, we have to fix the relative probabilities of the reference outcome (HU) to zero on the log scale
    beta[i, 1] <- 0
    # Loop over response outcomes
    for (j in 2:n.outcomes){
      # Assign diffuse priors to change (periods 1-2 and periods 1-3) in rel. probs. of outcomes
      beta[i, j] ~ dnorm(0, 0.001)   
      } 
  }
  
  # tau.j parameters represent inter-individual variation in relative probs. of outcomes
  # Loop over response outcomes
  for (j in 1:n.outcomes){  
    # Mean of individual random effects is 0
    mu.re[j] <- 0           
  }
  
  # PRIORS FOR ELEMENTS OF PRECISION MATRIX
  # ***********************************
  # df set to j+1
  prec[1:6,1:6] ~ dwish(R[,],7)  
  # Convert precision to covariance matrix
  sigma[1:6,1:6] <- inverse(prec[,])   
  # Correlation between outcome 1 and 2
  # rho <- sigma[1,2]/sqrt(sigma[1,1]*sigma[2,2])  
  
  # DEFINE INDIVIDUAL-LEVEL PARAMETERS
  # ***********************************
  # Loop over individuals to define individual-level random effects
  for (idx in 1:nind){  
    # Rel. prob. of reference outcome fixed to zero, so there is no adjustment among indiviuals
    eps[idx,1:6] ~ dmnorm(mu.re[], prec[,])      
  }
  
  # LIKELIHOOD 
  # ***********************************
  # Loop over observations
  for (i in 1:n) {     
    # Multinomial response
    Y[i, ] ~ dmulti(p[i, ] , N[i])

    # Loop through outcomes
    for (j in 1:n.outcomes) {     
      p[i,j] <- phi[i,j] / sum(phi[i, ])
      log(phi[i,j]) <- alpha[j] + beta[PERIOD[i], j] + eps[ind[i], j]
    }
  }
  
  # DERIVED QUANTITIES - Probabilities of each behavior during each period
  # ***********************************
  for (j in 1:n.outcomes){
    PROBS[1,j] <- PHI[1,j] / sum(PHI[1,])
    log(PHI[1,j]) <- alpha[j] + beta[1,j]
    PROBS[2,j] <- PHI[2,j] / sum(PHI[2,])
    log(PHI[2,j]) <- alpha[j] + beta[2,j]
    PROBS[3,j] <- PHI[3,j] / sum(PHI[3,])
    log(PHI[3,j]) <- alpha[j] + beta[3,j]
  }
}
```

### Fitting JAGS Model

Here we fit the model described above, specifying the parameters estimated to save in the output.

```
jm2=jags(model.file = "Model_Multinomial_withREs.R",
         data=data.list,
         n.chains=3,n.iter=n.iter,n.burnin = n.burnin,n.thin=n.thin,parallel = F,
         parameters.to.save = c("alpha","beta","sigma","PROBS","eps"))

# You will receive an error that "At least one Rhat value could not be calculated."  This is expected since these values were pre-set to zero (0) in our model above.
```

### Load Saved Model

Since running the model can take a lot of time, you can save the model as a `.Rda` file and load the model output from disk. The resulting object can then be loaded into R without going through all the steps to fit the model and prepare the dataframe.

```
# Save JAGS model
#save(jm2, file = "Behavior_Models.Rda")
load("Behavior_Models.Rda")

# Summarize object
jm2
```

```
## JAGS output for model 'Model_Multinomial_withREs.R', generated by jagsUI.
## Estimates based on 3 chains of 5e+05 iterations,
## adaptation = 600 iterations (sufficient),
## burn-in = 1e+05 iterations and thin rate = 100,
## yielding 12000 total samples from the joint posterior. 
## MCMC ran for 82.029 minutes at time 2018-12-16 20:51:50.
## 
##                mean     sd     2.5%      50%    97.5% overlap0     f  Rhat
## alpha[1]      0.000  0.000    0.000    0.000    0.000    FALSE 1.000    NA
## alpha[2]     -0.838  0.483   -1.765   -0.856    0.199     TRUE 0.952 1.011
## alpha[3]     -3.606  0.846   -5.281   -3.586   -1.952    FALSE 0.999 1.026
## alpha[4]     -2.211  0.175   -2.555   -2.211   -1.870    FALSE 1.000 1.000
## alpha[5]     -1.215  0.202   -1.627   -1.210   -0.823    FALSE 1.000 1.003
## alpha[6]     -1.535  0.227   -2.001   -1.532   -1.084    FALSE 1.000 1.006
## beta[1,1]     0.000  0.000    0.000    0.000    0.000    FALSE 1.000    NA
## beta[2,1]     0.000  0.000    0.000    0.000    0.000    FALSE 1.000    NA
## beta[3,1]     0.000  0.000    0.000    0.000    0.000    FALSE 1.000    NA
## beta[1,2]     0.000  0.000    0.000    0.000    0.000    FALSE 1.000    NA
## beta[2,2]     0.347  0.093    0.162    0.348    0.532    FALSE 1.000 1.000
## beta[3,2]     0.274  0.067    0.141    0.273    0.405    FALSE 1.000 1.000
## beta[1,3]     0.000  0.000    0.000    0.000    0.000    FALSE 1.000    NA
## beta[2,3]     0.831  0.163    0.517    0.831    1.150    FALSE 1.000 1.000
## beta[3,3]    -2.585  0.285   -3.169   -2.575   -2.044    FALSE 1.000 1.000
## beta[1,4]     0.000  0.000    0.000    0.000    0.000    FALSE 1.000    NA
## beta[2,4]     0.534  0.168    0.203    0.536    0.856    FALSE 0.999 1.000
## beta[3,4]    -0.600  0.149   -0.885   -0.602   -0.306    FALSE 1.000 1.000
## beta[1,5]     0.000  0.000    0.000    0.000    0.000    FALSE 1.000    NA
## beta[2,5]     0.284  0.115    0.060    0.284    0.507    FALSE 0.993 1.000
## beta[3,5]     0.359  0.086    0.192    0.361    0.527    FALSE 1.000 1.000
## beta[1,6]     0.000  0.000    0.000    0.000    0.000    FALSE 1.000    NA
## beta[2,6]    -0.821  0.183   -1.181   -0.820   -0.466    FALSE 1.000 1.000
## beta[3,6]    -0.942  0.123   -1.181   -0.942   -0.699    FALSE 1.000 1.001
## sigma[1,1]    0.516  0.708    0.054    0.299    2.360    FALSE 1.000 1.088
## sigma[2,1]    0.078  0.691   -0.823   -0.043    1.779     TRUE 0.452 1.084
## sigma[3,1]   -0.646  1.191   -3.509   -0.472    1.094     TRUE 0.800 1.071
## sigma[4,1]    0.417  0.703   -0.022    0.200    2.258     TRUE 0.938 1.107
## sigma[5,1]    0.551  0.818   -0.048    0.311    2.687     TRUE 0.924 1.077
## sigma[6,1]    0.393  0.710   -0.095    0.184    2.280     TRUE 0.846 1.089
## sigma[1,2]    0.078  0.691   -0.823   -0.043    1.779     TRUE 0.452 1.084
## sigma[2,2]    1.984  1.426    0.486    1.612    5.792    FALSE 1.000 1.010
## sigma[3,2]   -0.176  1.673   -3.372   -0.239    3.364     TRUE 0.598 1.027
## sigma[4,2]    0.280  0.755   -0.583    0.118    2.127     TRUE 0.634 1.084
## sigma[5,2]   -0.353  0.769   -1.685   -0.397    1.389     TRUE 0.810 1.070
## sigma[6,2]   -0.331  0.705   -1.494   -0.368    1.190     TRUE 0.824 1.076
## sigma[1,3]   -0.646  1.191   -3.509   -0.472    1.094     TRUE 0.800 1.071
## sigma[2,3]   -0.176  1.673   -3.372   -0.239    3.364     TRUE 0.598 1.027
## sigma[3,3]    4.728  4.090    0.887    3.590   15.443    FALSE 1.000 1.003
## sigma[4,3]   -0.719  1.220   -3.580   -0.539    0.992     TRUE 0.827 1.087
## sigma[5,3]   -0.913  1.357   -4.249   -0.675    0.977     TRUE 0.852 1.049
## sigma[6,3]   -0.349  1.276   -3.198   -0.239    1.717     TRUE 0.653 1.069
## sigma[1,4]    0.417  0.703   -0.022    0.200    2.258     TRUE 0.938 1.107
## sigma[2,4]    0.280  0.755   -0.583    0.118    2.127     TRUE 0.634 1.084
## sigma[3,4]   -0.719  1.220   -3.580   -0.539    0.992     TRUE 0.827 1.087
## sigma[4,4]    0.496  0.734    0.039    0.273    2.384    FALSE 1.000 1.119
## sigma[5,4]    0.444  0.805   -0.158    0.207    2.579     TRUE 0.799 1.092
## sigma[6,4]    0.408  0.716   -0.069    0.198    2.276     TRUE 0.892 1.107
## sigma[1,5]    0.551  0.818   -0.048    0.311    2.687     TRUE 0.924 1.077
## sigma[2,5]   -0.353  0.769   -1.685   -0.397    1.389     TRUE 0.810 1.070
## sigma[3,5]   -0.913  1.357   -4.249   -0.675    0.977     TRUE 0.852 1.049
## sigma[4,5]    0.444  0.805   -0.158    0.207    2.579     TRUE 0.799 1.092
## sigma[5,5]    0.962  1.004    0.129    0.668    3.588    FALSE 1.000 1.059
## sigma[6,5]    0.561  0.848   -0.108    0.320    2.838     TRUE 0.890 1.070
## sigma[1,6]    0.393  0.710   -0.095    0.184    2.280     TRUE 0.846 1.089
## sigma[2,6]   -0.331  0.705   -1.494   -0.368    1.190     TRUE 0.824 1.076
## sigma[3,6]   -0.349  1.276   -3.198   -0.239    1.717     TRUE 0.653 1.069
## sigma[4,6]    0.408  0.716   -0.069    0.198    2.276     TRUE 0.892 1.107
## sigma[5,6]    0.561  0.848   -0.108    0.320    2.838     TRUE 0.890 1.070
## sigma[6,6]    0.689  0.789    0.102    0.466    2.831    FALSE 1.000 1.071
## PROBS[1,1]    0.468  0.050    0.345    0.473    0.551    FALSE 1.000 1.016
## PROBS[2,1]    0.413  0.057    0.275    0.418    0.508    FALSE 1.000 1.019
## PROBS[3,1]    0.458  0.056    0.321    0.464    0.549    FALSE 1.000 1.012
## PROBS[1,2]    0.217  0.085    0.090    0.202    0.433    FALSE 1.000 1.013
## PROBS[2,2]    0.267  0.096    0.117    0.252    0.501    FALSE 1.000 1.010
## PROBS[3,2]    0.275  0.099    0.119    0.260    0.514    FALSE 1.000 1.013
## PROBS[1,3]    0.018  0.025    0.002    0.013    0.062    FALSE 1.000 1.210
## PROBS[2,3]    0.036  0.042    0.005    0.026    0.119    FALSE 1.000 1.177
## PROBS[3,3]    0.001  0.002    0.000    0.001    0.005    FALSE 1.000 1.231
## PROBS[1,4]    0.052  0.008    0.036    0.051    0.068    FALSE 1.000 1.008
## PROBS[2,4]    0.078  0.013    0.052    0.077    0.104    FALSE 1.000 1.014
## PROBS[3,4]    0.028  0.004    0.019    0.028    0.037    FALSE 1.000 1.005
## PROBS[1,5]    0.142  0.032    0.078    0.141    0.207    FALSE 1.000 1.008
## PROBS[2,5]    0.167  0.042    0.084    0.166    0.251    FALSE 1.000 1.010
## PROBS[3,5]    0.199  0.046    0.107    0.199    0.290    FALSE 1.000 1.009
## PROBS[1,6]    0.103  0.025    0.056    0.102    0.157    FALSE 1.000 1.010
## PROBS[2,6]    0.041  0.012    0.019    0.040    0.067    FALSE 1.000 1.009
## PROBS[3,6]    0.040  0.011    0.020    0.039    0.063    FALSE 1.000 1.010
## eps[1,1]     -0.301  0.466   -1.306   -0.284    0.564     TRUE 0.745 1.010
## eps[2,1]     -0.346  0.384   -1.137   -0.344    0.432     TRUE 0.840 1.012
## eps[3,1]      0.632  0.475   -0.141    0.566    1.770     TRUE 0.949 1.028
## eps[4,1]     -0.294  0.631   -1.637   -0.282    0.929     TRUE 0.688 1.006
## eps[5,1]     -0.398  0.312   -1.072   -0.383    0.200     TRUE 0.916 1.007
## eps[6,1]      0.042  0.335   -0.567    0.017    0.796     TRUE 0.525 1.051
## eps[7,1]     -0.101  0.542   -0.917   -0.180    1.265     TRUE 0.680 1.149
## eps[8,1]      0.110  0.567   -0.782    0.045    1.379     TRUE 0.545 1.099
## eps[9,1]      1.102  0.977   -0.549    0.983    3.352     TRUE 0.905 1.038
## eps[10,1]    -0.045  0.434   -0.981   -0.028    0.772     TRUE 0.526 1.011
## eps[1,2]      0.144  0.683   -1.284    0.152    1.504     TRUE 0.590 1.020
## eps[2,2]      0.954  0.604   -0.324    0.967    2.142     TRUE 0.942 1.010
## eps[3,2]      0.615  0.573   -0.440    0.575    1.837     TRUE 0.878 1.007
## eps[4,2]     -2.472  0.782   -4.220   -2.427   -1.025    FALSE 1.000 1.014
## eps[5,2]      0.146  0.552   -1.059    0.163    1.226     TRUE 0.624 1.014
## eps[6,2]      0.878  0.506   -0.128    0.860    1.901     TRUE 0.961 1.008
## eps[7,2]      0.904  0.636   -0.180    0.840    2.317     TRUE 0.952 1.063
## eps[8,2]      1.003  0.655   -0.104    0.935    2.400     TRUE 0.963 1.044
## eps[9,2]     -2.244  1.023   -4.250   -2.271   -0.082    FALSE 0.977 1.020
## eps[10,2]     0.127  0.657   -1.244    0.135    1.442     TRUE 0.590 1.019
## eps[1,3]      2.272  0.877    0.640    2.207    4.116    FALSE 0.992 1.030
## eps[2,3]      1.018  0.810   -0.538    0.980    2.717     TRUE 0.908 1.018
## eps[3,3]     -1.854  1.132   -4.453   -1.721   -0.038    FALSE 0.977 1.009
## eps[4,3]      2.036  0.962    0.130    1.991    4.000    FALSE 0.980 1.022
## eps[5,3]      1.254  0.783   -0.186    1.196    2.917     TRUE 0.959 1.023
## eps[6,3]     -0.705  0.850   -2.484   -0.671    0.941     TRUE 0.808 1.003
## eps[7,3]     -1.574  1.223   -4.656   -1.407    0.337     TRUE 0.942 1.013
## eps[8,3]     -1.849  1.229   -4.734   -1.671    0.006     TRUE 0.974 1.010
## eps[9,3]     -2.824  2.079   -8.081   -2.471    0.250     TRUE 0.960 1.006
## eps[10,3]     2.065  0.858    0.483    2.008    3.868    FALSE 0.990 1.030
## eps[1,4]     -0.429  0.487   -1.490   -0.409    0.480     TRUE 0.822 1.011
## eps[2,4]     -0.530  0.418   -1.392   -0.522    0.288     TRUE 0.912 1.012
## eps[3,4]      0.250  0.482   -0.535    0.182    1.420     TRUE 0.690 1.032
## eps[4,4]     -0.288  0.646   -1.661   -0.280    0.950     TRUE 0.678 1.005
## eps[5,4]     -0.174  0.332   -0.896   -0.157    0.446     TRUE 0.709 1.010
## eps[6,4]      0.304  0.346   -0.284    0.272    1.104     TRUE 0.838 1.057
## eps[7,4]      0.360  0.562   -0.429    0.270    1.744     TRUE 0.785 1.153
## eps[8,4]      0.610  0.584   -0.248    0.531    1.938     TRUE 0.919 1.107
## eps[9,4]      0.591  0.989   -1.076    0.470    2.910     TRUE 0.732 1.036
## eps[10,4]    -0.324  0.458   -1.304   -0.304    0.533     TRUE 0.766 1.011
## eps[1,5]     -0.971  0.486   -2.010   -0.943   -0.086    FALSE 0.984 1.006
## eps[2,5]      0.204  0.416   -0.626    0.205    1.039     TRUE 0.702 1.012
## eps[3,5]      0.295  0.528   -0.606    0.240    1.509     TRUE 0.710 1.027
## eps[4,5]      0.383  0.663   -0.987    0.373    1.717     TRUE 0.741 1.004
## eps[5,5]     -0.229  0.354   -0.962   -0.215    0.440     TRUE 0.754 1.004
## eps[6,5]     -0.231  0.392   -0.955   -0.247    0.602     TRUE 0.756 1.045
## eps[7,5]      0.166  0.591   -0.762    0.091    1.647     TRUE 0.593 1.132
## eps[8,5]     -0.270  0.608   -1.255   -0.326    1.057     TRUE 0.744 1.091
## eps[9,5]      1.991  1.023    0.273    1.884    4.376    FALSE 0.990 1.036
## eps[10,5]    -0.860  0.458   -1.844   -0.836   -0.010    FALSE 0.976 1.006
## eps[1,6]     -0.189  0.487   -1.221   -0.179    0.729     TRUE 0.652 1.006
## eps[2,6]     -0.966  0.424   -1.852   -0.947   -0.157    FALSE 0.988 1.014
## eps[3,6]     -0.080  0.515   -0.981   -0.127    1.101     TRUE 0.614 1.032
## eps[4,6]      1.051  0.658   -0.290    1.048    2.381     TRUE 0.944 1.003
## eps[5,6]     -0.238  0.344   -0.955   -0.234    0.420     TRUE 0.764 1.008
## eps[6,6]     -0.219  0.378   -0.955   -0.231    0.553     TRUE 0.756 1.050
## eps[7,6]      0.313  0.583   -0.613    0.241    1.736     TRUE 0.741 1.134
## eps[8,6]      0.325  0.605   -0.646    0.266    1.581     TRUE 0.740 1.096
## eps[9,6]      0.912  1.007   -0.808    0.802    3.220     TRUE 0.846 1.037
## eps[10,6]    -0.485  0.462   -1.463   -0.469    0.390     TRUE 0.860 1.007
## deviance   8143.383 12.152 8121.452 8142.695 8169.146    FALSE 1.000 1.000
##            n.eff
## alpha[1]       1
## alpha[2]     230
## alpha[3]     145
## alpha[4]    6140
## alpha[5]     732
## alpha[6]     372
## beta[1,1]      1
## beta[2,1]      1
## beta[3,1]      1
## beta[1,2]      1
## beta[2,2]   7000
## beta[3,2]  12000
## beta[1,3]      1
## beta[2,3]  12000
## beta[3,3]  12000
## beta[1,4]      1
## beta[2,4]  12000
## beta[3,4]  12000
## beta[1,5]      1
## beta[2,5]  12000
## beta[3,5]  12000
## beta[1,6]      1
## beta[2,6]  12000
## beta[3,6]   7235
## sigma[1,1]    99
## sigma[2,1]    97
## sigma[3,1]   104
## sigma[4,1]    82
## sigma[5,1]   114
## sigma[6,1]   110
## sigma[1,2]    97
## sigma[2,2]   345
## sigma[3,2]   246
## sigma[4,2]   100
## sigma[5,2]    98
## sigma[6,2]   125
## sigma[1,3]   104
## sigma[2,3]   246
## sigma[3,3]  1825
## sigma[4,3]   109
## sigma[5,3]   128
## sigma[6,3]   109
## sigma[1,4]    82
## sigma[2,4]   100
## sigma[3,4]   109
## sigma[4,4]    76
## sigma[5,4]    92
## sigma[6,4]    92
## sigma[1,5]   114
## sigma[2,5]    98
## sigma[3,5]   128
## sigma[4,5]    92
## sigma[5,5]   151
## sigma[6,5]   131
## sigma[1,6]   110
## sigma[2,6]   125
## sigma[3,6]   109
## sigma[4,6]    92
## sigma[5,6]   131
## sigma[6,6]   143
## PROBS[1,1]   241
## PROBS[2,1]   188
## PROBS[3,1]   322
## PROBS[1,2]   246
## PROBS[2,2]   281
## PROBS[3,2]   214
## PROBS[1,3]    91
## PROBS[2,3]    93
## PROBS[3,3]   104
## PROBS[1,4]   411
## PROBS[2,4]   235
## PROBS[3,4]   488
## PROBS[1,5]   271
## PROBS[2,5]   215
## PROBS[3,5]   259
## PROBS[1,6]   203
## PROBS[2,6]   225
## PROBS[3,6]   205
## eps[1,1]     545
## eps[2,1]     507
## eps[3,1]     121
## eps[4,1]     419
## eps[5,1]   12000
## eps[6,1]      62
## eps[7,1]      37
## eps[8,1]      50
## eps[9,1]     187
## eps[10,1]    732
## eps[1,2]     179
## eps[2,2]    1551
## eps[3,2]     552
## eps[4,2]     168
## eps[5,2]     369
## eps[6,2]     873
## eps[7,2]      89
## eps[8,2]     124
## eps[9,2]     358
## eps[10,2]    203
## eps[1,3]      98
## eps[2,3]     153
## eps[3,3]     225
## eps[4,3]     108
## eps[5,3]     118
## eps[6,3]     607
## eps[7,3]     244
## eps[8,3]     394
## eps[9,3]     360
## eps[10,3]     99
## eps[1,4]     609
## eps[2,4]     529
## eps[3,4]     114
## eps[4,4]     429
## eps[5,4]   10354
## eps[6,4]      60
## eps[7,4]      37
## eps[8,4]      50
## eps[9,4]     180
## eps[10,4]    844
## eps[1,5]    1127
## eps[2,5]     291
## eps[3,5]     115
## eps[4,5]     860
## eps[5,5]    1128
## eps[6,5]      64
## eps[7,5]      39
## eps[8,5]      49
## eps[9,5]     172
## eps[10,5]   1158
## eps[1,6]    1388
## eps[2,6]     194
## eps[3,6]      91
## eps[4,6]    1056
## eps[5,6]     478
## eps[6,6]      56
## eps[7,6]      37
## eps[8,6]      48
## eps[9,6]     166
## eps[10,6]   1187
## deviance    6698
## 
## **WARNING** Rhat values indicate convergence failure. 
## Rhat is the potential scale reduction factor (at convergence, Rhat=1). 
## For each parameter, n.eff is a crude measure of effective sample size. 
## 
## overlap0 checks if 0 falls in the parameter's 95% credible interval.
## f is the proportion of the posterior with the same sign as the mean;
## i.e., our confidence that the parameter is positive or negative.
## 
## DIC info: (pD = var(deviance)/2) 
## pD = 73.8 and DIC = 8217.208 
## DIC is an estimate of expected predictive error (lower is better).
```

### Summarize Results

Summarize and view the parameters estimated by the model, calculating the highest posterior density intervals. Plot the probabilities of each behavior to assess model convergence. Important in examining the results is keeping track of each parameter, as the parameters are annotated in the output matrix and dependent on the order in which they were input.

```
# eps are the individual random effects
# tau.j's are the random effects for each behavior

# Investigate values in output
jm2$mean
```

```
## $alpha
## [1]  0.0000000 -0.8383666 -3.6057551 -2.2111849 -1.2153529 -1.5345287
## 
## $beta
##      [,1]      [,2]       [,3]       [,4]      [,5]       [,6]
## [1,]    0 0.0000000  0.0000000  0.0000000 0.0000000  0.0000000
## [2,]    0 0.3472518  0.8308967  0.5337492 0.2839093 -0.8212494
## [3,]    0 0.2736763 -2.5846088 -0.6000964 0.3594307 -0.9419157
## 
## $sigma
##             [,1]        [,2]       [,3]       [,4]       [,5]       [,6]
## [1,]  0.51596268  0.07845459 -0.6464594  0.4166945  0.5508934  0.3934505
## [2,]  0.07845459  1.98428366 -0.1761951  0.2803716 -0.3525543 -0.3305368
## [3,] -0.64645936 -0.17619507  4.7283600 -0.7194287 -0.9130762 -0.3494501
## [4,]  0.41669452  0.28037159 -0.7194287  0.4961227  0.4437328  0.4078545
## [5,]  0.55089338 -0.35255432 -0.9130762  0.4437328  0.9617223  0.5613727
## [6,]  0.39345050 -0.33053680 -0.3494501  0.4078545  0.5613727  0.6886885
## 
## $PROBS
##           [,1]      [,2]        [,3]       [,4]      [,5]       [,6]
## [1,] 0.4681135 0.2168090 0.018326837 0.05158935 0.1418437 0.10331764
## [2,] 0.4128297 0.2666037 0.035925363 0.07750826 0.1665949 0.04053807
## [3,] 0.4576258 0.2751442 0.001443507 0.02763890 0.1985519 0.03959574
## 
## $eps
##              [,1]       [,2]       [,3]       [,4]       [,5]        [,6]
##  [1,] -0.30113252  0.1439376  2.2718239 -0.4293615 -0.9713507 -0.18895089
##  [2,] -0.34613870  0.9543164  1.0184830 -0.5296255  0.2043327 -0.96593344
##  [3,]  0.63206329  0.6149179 -1.8540194  0.2496119  0.2952365 -0.07967988
##  [4,] -0.29380316 -2.4715177  2.0361184 -0.2883281  0.3827409  1.05062742
##  [5,] -0.39835708  0.1456536  1.2539847 -0.1737483 -0.2293806 -0.23774199
##  [6,]  0.04163115  0.8777452 -0.7045506  0.3037302 -0.2309182 -0.21919314
##  [7,] -0.10133551  0.9036653 -1.5740339  0.3597239  0.1662376  0.31322408
##  [8,]  0.11011639  1.0029491 -1.8489093  0.6095973 -0.2698393  0.32497887
##  [9,]  1.10213881 -2.2444181 -2.8244698  0.5909258  1.9913290  0.91204110
## [10,] -0.04504055  0.1268903  2.0652818 -0.3241115 -0.8604195 -0.48491780
## 
## $deviance
## [1] 8143.383
```

```
# Look at trace and density plots to assess model convergence
MCMCtrace(jm2, params = 'PROBS', ind=TRUE, pdf=FALSE)
```

```
# Summarize values from the output, include median and highest posterior density intervals
Post.Summary <- MCMCsummary(jm2, 
                            params = 'PROBS',
                            Rhat = TRUE,
                            n.eff = TRUE,
                            func = function(x) c(median(x), hdi(x,credMass = 0.95)),
                            func_name = c('median','hdi_low','hdi_high'))

# View
Post.Summary
```

```
##                   mean          sd         2.5%          50%       97.5%
## PROBS[1,1] 0.468113507 0.050135112 0.3452597673 0.4734731573 0.550999899
## PROBS[2,1] 0.412829706 0.056517746 0.2745183875 0.4184394258 0.508337790
## PROBS[3,1] 0.457625815 0.055933091 0.3212907639 0.4643137082 0.549171356
## PROBS[1,2] 0.216809019 0.085196304 0.0895645411 0.2020092299 0.432806209
## PROBS[2,2] 0.266603657 0.095630551 0.1174171623 0.2521404296 0.501278263
## PROBS[3,2] 0.275144186 0.098604799 0.1191237348 0.2603164587 0.514106643
## PROBS[1,3] 0.018326837 0.024946374 0.0023479532 0.0129520502 0.061618395
## PROBS[2,3] 0.035925363 0.042428739 0.0048273645 0.0262523740 0.118965229
## PROBS[3,3] 0.001443507 0.002449803 0.0001620766 0.0009441542 0.005008333
## PROBS[1,4] 0.051589352 0.007901954 0.0364024338 0.0512907991 0.068137846
## PROBS[2,4] 0.077508261 0.013134097 0.0518954936 0.0772909189 0.103976892
## PROBS[3,4] 0.027638896 0.004363375 0.0192060030 0.0275097856 0.036824139
## PROBS[1,5] 0.141843650 0.032305607 0.0783893372 0.1414830557 0.207005914
## PROBS[2,5] 0.166594943 0.042005857 0.0844023524 0.1660314484 0.251141983
## PROBS[3,5] 0.198551854 0.046000670 0.1068223736 0.1988336212 0.289789908
## PROBS[1,6] 0.103317636 0.024623812 0.0560820141 0.1022779477 0.157068763
## PROBS[2,6] 0.040538070 0.011950662 0.0192955049 0.0396090680 0.066787881
## PROBS[3,6] 0.039595742 0.010610692 0.0201969014 0.0390113913 0.063077288
##            Rhat n.eff       median      hdi_low    hdi_high
## PROBS[1,1] 1.01   350 0.4734731573 3.542915e-01 0.555413205
## PROBS[2,1] 1.02   281 0.4184394258 2.952800e-01 0.521764882
## PROBS[3,1] 1.01   280 0.4643137082 3.402847e-01 0.560328977
## PROBS[1,2] 1.02   199 0.2020092299 6.536400e-02 0.383609795
## PROBS[2,2] 1.02   198 0.2521404296 9.038427e-02 0.456527520
## PROBS[3,2] 1.01   196 0.2603164587 9.965903e-02 0.477034804
## PROBS[1,3] 1.25   206 0.0129520502 8.577084e-04 0.044343423
## PROBS[2,3] 1.22   188 0.0262523740 1.957731e-03 0.088991221
## PROBS[3,3] 1.26   235 0.0009441542 5.231638e-05 0.003625889
## PROBS[1,4] 1.01  2045 0.0512907991 3.562382e-02 0.067180779
## PROBS[2,4] 1.02  1255 0.0772909189 5.141142e-02 0.103370081
## PROBS[3,4] 1.00  1566 0.0275097856 1.902358e-02 0.036545012
## PROBS[1,5] 1.01   330 0.1414830557 7.404103e-02 0.201555545
## PROBS[2,5] 1.01   270 0.1660314484 8.249655e-02 0.248447807
## PROBS[3,5] 1.01   267 0.1988336212 1.027117e-01 0.284509023
## PROBS[1,6] 1.01   428 0.1022779477 5.459032e-02 0.154456761
## PROBS[2,6] 1.01   452 0.0396090680 1.752259e-02 0.064017782
## PROBS[3,6] 1.01   368 0.0390113913 1.781983e-02 0.060169798
```

### Export Summary

Output `Post.Summary` to re-create Appendix S5.

```
# Export file
write.csv(Post.Summary, "./Output/jm2_Output_Summary.csv")
```

### Plot Results

Using the `MCMCplot` function, plot the probabilities of the behaviors with significant effects from the pre-treatment period. Again, here, it is important to keep track of the parameters output from the model. Note that the reference category (pre-treatment period) has been set to ‘0’. Thus, treatment and post-treatment periods are the probability of behavior in reference to this pre-treatment period. This allowed us to evaluate the general effect of collaring on each behavior. The code could be easily modified to plot all variables estimated. To save this plot to a directory, the command `png()` and `dev.off()` can be activated in the code.

```
# Plot the caterpillar plots for the entire MCMC output
#MCMCplot(jm2, params = 'PROBS')

# Set all the Labels
main.label <- c("Head-Up", "Head-Down", "Laying", "Headshaking", "Locomotion", "Scratching")

# Display only the graphs where a significant change occurred
#png(file = "./Output/PROBS_variables.png",width=15, height=5, units = 'in', res=500)
layout(matrix(c(1,2,3), 1, 3, byrow = FALSE), widths=1, heights=1)

MCMCplot(jm2, params = c('PROBS\\[1,3\\]', 'PROBS\\[2,3\\]', 'PROBS\\[3,3\\]'), ref = Post.Summary[7,8], 
         ref_ovl = TRUE, ISB=FALSE, 
         main=main.label[3],
         med_sz=1.5, thin_sz = 1, thick_sz = 3, ax_sz=2, main_text_sz=2,axis_text_sz=1.5,tick_text_sz = 1.5, 
         labels_sz = 2,
         labels=c('Pre-Trmt','Trmt','Post-Trmt'), xlab="Probability",
         mar = c(5.1, 6.1, 4.1, 2.1))
MCMCplot(jm2, params = c('PROBS\\[1,4\\]', 'PROBS\\[2,4\\]', 'PROBS\\[3,4\\]'), ref = Post.Summary[10,8], 
         ref_ovl = TRUE, ISB=FALSE, 
         main=main.label[4],
         med_sz=1.5, thin_sz = 1, thick_sz = 3, ax_sz=2, main_text_sz=2,axis_text_sz=1.5,tick_text_sz = 1.5, 
         labels=NULL, xlab="Probability",
         mar = c(5.1, 6.1, 4.1, 2.1))
MCMCplot(jm2, params = c('PROBS\\[1,6\\]', 'PROBS\\[2,6\\]', 'PROBS\\[3,6\\]'), ref = Post.Summary[16,8], 
         ref_ovl = TRUE, ISB=FALSE, 
         main=main.label[6],
         med_sz=1.5, thin_sz = 1, thick_sz = 3, ax_sz=2, main_text_sz=2,axis_text_sz=1.5,tick_text_sz = 1.5, 
         labels=NULL, xlab="Probability")
```

```
#dev.off()
```
