# Supplementary figures and images for: Short-term effects of GPS collars on the activity, behavior, and adrenal response of scimitar-horned oryx (Oryx dammah)
Source: PLoS One. 2020 Feb 11;15(2):e0221843. doi: 10.1371/journal.pone.0221843 (PMC7012457; doi:10.1371/journal.pone.0221843)

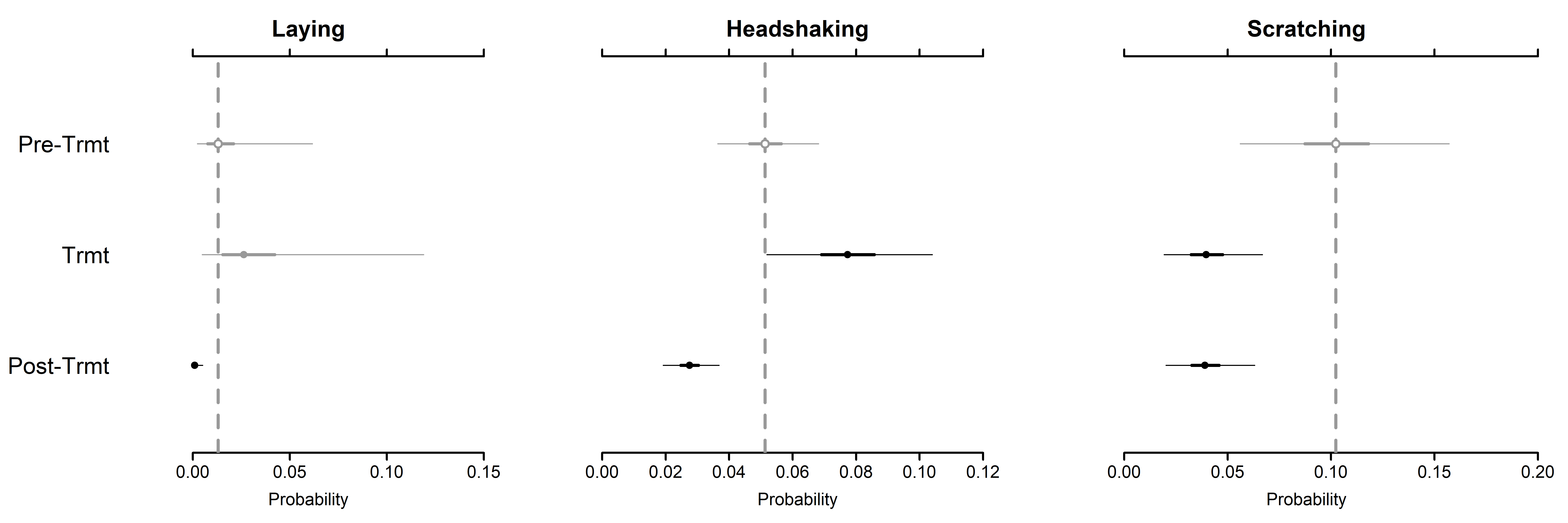

Supplement: S1 Code — Script and data tutorial to investigate behavioral changes observed in scimitar-horned oryx (Oryx dammah) fit with GPS collars. Data fit in a Bayesian framework, estimating the probability of each behavioral activity and based on a multinomial likelihood. Each animal was used as their own control to assess how each behavior changed across time periods (Pre-treatment, Treatment, Post-treatment). (ZIP) [file pone.0221843.s001.zip › S1_Code/Output/PROBS_variables.png]

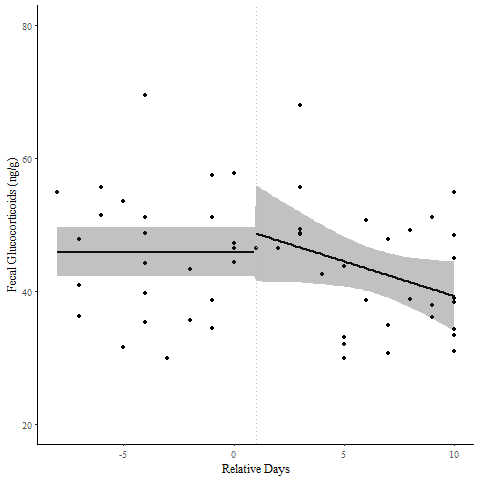

Supplement: S2 Code — Script and data tutorial to investigate changes in fecal glucocorticoid metabolite levels in scimitar-horned oryx (Oryx dammah). Data fit in a Bayesian framework, testing various piecewise regression models to predict the response of animals fitted with GPS collars. Data split between treatment (collared) and control (non-collared) animals. (ZIP) [file pone.0221843.s002.zip › S2_Code/Output/Habituation.png]

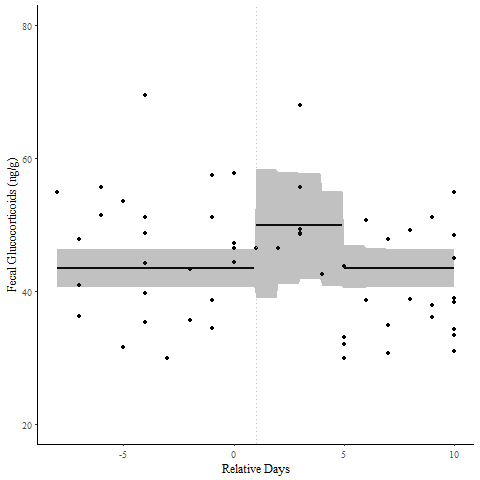

Supplement: S2 Code — Script and data tutorial to investigate changes in fecal glucocorticoid metabolite levels in scimitar-horned oryx (Oryx dammah). Data fit in a Bayesian framework, testing various piecewise regression models to predict the response of animals fitted with GPS collars. Data split between treatment (collared) and control (non-collared) animals. (ZIP) [file pone.0221843.s002.zip › S2_Code/Output/Handling.png]

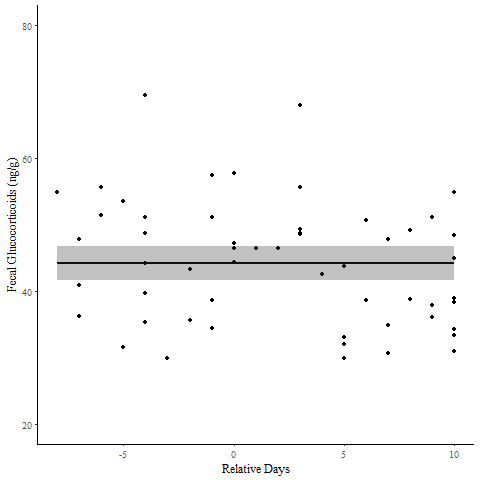

Supplement: S2 Code — Script and data tutorial to investigate changes in fecal glucocorticoid metabolite levels in scimitar-horned oryx (Oryx dammah). Data fit in a Bayesian framework, testing various piecewise regression models to predict the response of animals fitted with GPS collars. Data split between treatment (collared) and control (non-collared) animals. (ZIP) [file pone.0221843.s002.zip › S2_Code/Output/NoStress.png]

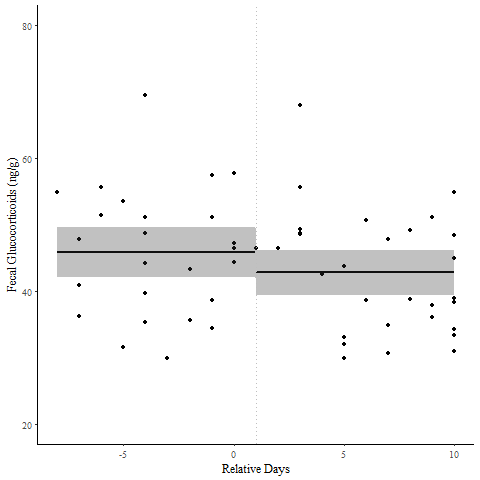

Supplement: S2 Code — Script and data tutorial to investigate changes in fecal glucocorticoid metabolite levels in scimitar-horned oryx (Oryx dammah). Data fit in a Bayesian framework, testing various piecewise regression models to predict the response of animals fitted with GPS collars. Data split between treatment (collared) and control (non-collared) animals. (ZIP) [file pone.0221843.s002.zip › S2_Code/Output/Stress.png]
