# Supplementary material for: Short-term effects of GPS collars on the activity, behavior, and adrenal response of scimitar-horned oryx (Oryx dammah)
Source: PLoS One. 2020 Feb 11;15(2):e0221843. doi: 10.1371/journal.pone.0221843 (PMC7012457; doi:10.1371/journal.pone.0221843)
Supplement: S2 Code — Script and data tutorial to investigate changes in fecal glucocorticoid metabolite levels in scimitar-horned oryx (Oryx dammah). Data fit in a Bayesian framework, testing various piecewise regression models to predict the response of animals fitted with GPS collars. Data split between treatment (collared) and control (non-collared) animals. (ZIP) [file pone.0221843.s002.zip › S2_Code/FGM_Analysis_PiecewiseRegression.html]

Piece-wise Regression Analysis: Fecal Glucocorticoid Metabolite Data


# Piece-wise Regression Analysis: Fecal Glucocorticoid Metabolite Data

### *Stabach et al. 2019 - Effects of GPS Collars*

#### *Jared Stabach, Smithsonian Conservation Biology Institute*

#### *2019-December-18*

# Piecewise Regression

> Comments/Questions: Contact Grant Connette (connetteg@si.edu) and Jared Stabach (stabachj@si.edu)

Data analysis to investigate changes in fecal glucocorticoid metabolite levels in scimitar-horned oryx (*Oryx dammah*). Data fit in a Bayesian framework, testing various piecewise regression models to predict the response of animals fitted with GPS collars. Data split between treatment (collared) and control (non-collared) animals.

The steps described here are aimed at recreating the figures and tables included in Stabach et al. 2019. Importantly, this includes recreating Figure 2 and determining which of the four models best fits the data. In the figure below, **A** represents the adrenal response of treatment animals fitted with GPS collars and **B** represents the adrenal response animals that were not fitted with a GPS collar (control animals).

Additional details can be found in:

Stabach, J.A., S.A. Cunningham, G. Connette, J.L. Mota, D. Reed, M. Byron, M. Songer, T. Wacher, K. Mertes, J.L. Brown, P. Comizzoli, J. Newby, S. Monfort, and P. Leimgruber. In Review. Short-term effects of GPS collars on the behavior and stress of scimitar-horned oryx (*Oryx dammah*). PlosOne.

### Load Libraries

Load each library necessary to complete the analysis.

```
# Clear objects in memory
rm(list=ls())

# Load library
library(jagsUI)
library(ggplot2)
library(MCMCvis)
library(HDInterval)
```

### Read/Prepare Data

Import the dataframe into R, subsetting the data into two different groups (collar and control).

```
# Import file (or use file.choose())
stress <- read.csv("./Data/long.stress.values2.csv", stringsAsFactors=FALSE)

# Exclude Bamako (no data) and get rid of the lines with NAs
stress <- subset(stress, Animal!="Bamako")
stress <- stress[!(is.na(stress$fGC)),]

# Look at data
head(stress)
```

```
##    RelDay Phase Animal   fGC   Group
## 28      0  post    Cat 38.22 control
## 31      3  post    Cat 43.47 control
## 32      4  post    Cat 49.88 control
## 34      6  post    Cat 32.34 control
## 50      3  post  Chari 48.69  collar
## 52      5  post  Chari 33.04  collar
```

### Subset Data

Subset data into Control and Treatment groups. Summarize the number of records from each group.

```
# Subset Groups
collar <- subset(stress, Group=="collar")
control <- subset(stress, Group=="control" | Group=="baseline")

# How many of each group
nrow(collar);nrow(control)
```

```
## [1] 55
```

```
## [1] 21
```

```
# Which animals are included in each group
unique(collar$Animal);length(unique(collar$Animal))
```

```
## [1] "Chari"  "Jena"   "Lewis"  "Niamey" "Ruby"   "Scout"  "Shadow" "Tex"   
## [9] "Violet"
```

```
## [1] 9
```

```
unique(control$Animal);length(unique(control$Animal))
```

```
## [1] "Cat"     "DrBob"   "Loretta"
```

```
## [1] 3
```

### Data Analysis

Here we analyze the Treatment data (Collared animals), fitting four piecewise regression models to evaluate a variety of potential responses of oryx to GPS collar fitting. These same steps could then be duplicated with the ‘Control’ data to ultimately create the graph displayed above. We calculate WAIC as a model selection criterion for each model, but ultimately use leave-one-out cross-validation to compare model support based on the sum of squared errors. First, however, we fit the data to each model.

First, we setup the number of iterations, burn-in, and thinning rate. We ran three paralle Markov chain Monte Carlo (MCMC) chains. Note, the number of iterations (`n.iter`) has been reduced (e.g., 200,000) so that analyses will execute quickly. `n.iter` should be increased in subsequent analyses to make sure the entirety of the parameter space has been thoroughly explored.

For results presented in our manuscript, we ran for 400,000 iterations, discarding the first 80,000 iterations (20%) of each chain as burn-in, and thinned the remaining posterior samples at a rate of 1:100. The joint posterior distribution, therefore, consisted of a total of 9,600 samples. Convergence was assessed by visual inspection of traceplots to ensure a reasonable exploration of the parameter space and by ensuring that the potential scale reduction factor was <1.1 for each variable (Gelman & Rubin 1992).

For each model, we assigned diffuse \(Uniform(0,100)\) priors for all \(\beta\_0\) coefficients, representing initial FGM concentrations in each model. \(\beta\_1\) coefficients were assigned \(Uniform(-50,50)\) priors, representing post-collaring shifts in FGM levels. We assigned \(Uniform(-10,10)\) priors to the \(\beta\_2\) coefficient, representing the per-day change in FGM levels after the breakpoint in the habituation response model. In the Handling Reponse model, we used a continuous parameterization for the breakpoint parameter, \(k\_2\), indicating the day when FGM levels were no longer elevated after handling, assuming this breakpoint had an equal prior probability of occurring on days 2 through 10.

```
# Set-up burn-in/iterations for JAGS
n.iter <- 200000 # Number of iterations.  
n.burnin <- n.iter*0.20 # burn-in iterations (0.20 percent)
n.thin <- 100
```

### Model 1: “No Response” Hypothesis

Our first model represents our hypothesis that stress levels remain unchanged throughout the study period. This is an intercept only model (simple linear regression with an intercept but no slope) and is formulated as:  
\[y\_i = \beta\_0 + \varepsilon\_i\] \[\varepsilon\_i \sim Normal(0, \sigma^2)\]

where FGM levels at each observation, \(i\), were treated as realizations of a normal distributions with mean \(\beta\_0\) and inter-observation variance parameter \(\sigma^2\).

We save this model as `Model1_Linear.R` so that it can be sourced in **JAGS**. We expect this model to look similar to:


The model parameterization is:

```
# Simple linear model with an intercept and no slope
model {
    
    # Priors
    b0 ~ dunif(0,100)
    sd ~ dunif(0,25)
    tau <- 1/pow(sd,2)
    
    # Likelihood
    for (i in 1:nobs) {
      y[i] ~ dnorm(b0, tau)
    }
}
```

#### Model 1: Fit

We create a data list, define the parameters we want reported from the model, run the model in **JAGS**, and summarize results.

```
# Create Data List
data <- list(
  nobs = nrow(collar), #Total number of observations
  y = collar$fGC #FGC measurements at each observation
)

# Define parameters
params <- c('b0','sd')

# Execute jags model
mod1 <- jags(data = data, inits = NULL, parameters.to.save = params, 
             model.file = "Model1_Linear.R", n.chains = 3, n.iter = n.iter, n.burnin = n.burnin, n.thin = n.thin, parallel=T)
```

```
## 
## Processing function input....... 
## 
## Done. 
##  
## Beginning parallel processing using 3 cores. Console output will be suppressed.
## 
## Parallel processing completed.
## 
## Calculating statistics....... 
## 
## Done.
```

```
print(mod1,3)
```

```
## JAGS output for model 'Model1_Linear.R', generated by jagsUI.
## Estimates based on 3 chains of 2e+05 iterations,
## adaptation = 100 iterations (sufficient),
## burn-in = 40000 iterations and thin rate = 100,
## yielding 4800 total samples from the joint posterior. 
## MCMC ran in parallel for 0.196 minutes at time 2019-12-18 21:12:30.
## 
##             mean    sd    2.5%     50%   97.5% overlap0 f Rhat n.eff
## b0        44.196 1.294  41.673  44.201  46.727    FALSE 1    1  4035
## sd         9.542 0.957   7.943   9.460  11.672    FALSE 1    1  4800
## deviance 402.480 2.124 400.436 401.837 408.282    FALSE 1    1  4800
## 
## Successful convergence based on Rhat values (all < 1.1). 
## Rhat is the potential scale reduction factor (at convergence, Rhat=1). 
## For each parameter, n.eff is a crude measure of effective sample size. 
## 
## overlap0 checks if 0 falls in the parameter's 95% credible interval.
## f is the proportion of the posterior with the same sign as the mean;
## i.e., our confidence that the parameter is positive or negative.
## 
## DIC info: (pD = var(deviance)/2) 
## pD = 2.3 and DIC = 404.736 
## DIC is an estimate of expected predictive error (lower is better).
```

#### Model 1: Summarize

Summarize and view results. Calculate the highest posterior density intervals.

```
# Look at trace and density plots to assess model convergence
MCMCtrace(mod1, ind=TRUE, pdf=FALSE)
```

```
# Summarize results.  Include median
Post.Summary <- MCMCsummary(mod1, 
                            params = c('b0'),
                            Rhat = TRUE,
                            n.eff = TRUE,
                            func = function(x) c(median(x),hdi(x,credMass = 0.95)),
                            func_name = c('median','hdi_low','hdi_high'))

# View result summary
Post.Summary
```

```
##        mean       sd     2.5%      50%    97.5% Rhat n.eff   median
## b0 44.19639 1.294181 41.67305 44.20148 46.72741    1  4396 44.20148
##     hdi_low hdi_high
## b0 41.50043 46.55018
```

```
# Export file
write.csv(Post.Summary, "./Output/Control/Model_NoStress.csv")
```

#### Model 1: WAIC

Calculate WAIC.

```
y = collar$fGC
lik <- loglik <- matrix(NA, nrow = length(mod1$sims.list$b0), ncol = nrow(collar))

# Likelihood of observed fGC value given the b0 and standard deviation estimates
for (i in 1:nrow(collar)){
  lik[,i] <- dnorm(y[i], mean = mod1$sims.list$b0, sd=mod1$sims.list$sd)
  loglik[,i] <- log(lik[,i])
}

# Calculate WAIC
lppd <- sum(log(apply(lik,2,mean))) #Sum the log pointwise posterior predictive densities
pWAIC1 <- 2 * sum(log(apply(lik,2,mean)) - apply(loglik,2,mean)) #Not used
pWAIC2 <- sum(apply(loglik,2,var)) #Posterior variance of log pointwise predictive densities
elppd2 <- lppd-pWAIC2
(WAIC <- -2*elppd2) # Calculate and print model WAIC
```

```
## [1] 404.4887
```

#### Model 1: Plot

To save this plot to a directory, the command `png()` and `dev.off()` can be activated in the code below.

```
# Setup x|y axes
Time <- -8:10
Vpred <- rep(mod1$mean$b0, length(Time))

lCI <- rep(quantile(mod1$sims.list$b0, probs=c(0.025)), length(Time))
UCI <- rep(quantile(mod1$sims.list$b0, probs=c(0.975)), length(Time))

# Plot using GGPlot
Output <- data.frame(x = Time,
                     y = Vpred,
                     lCI = lCI,
                     UCI = UCI)

#png("./Output/NoStress.png") # Activate if what to output to png
ggplot(collar) +
  geom_point(aes(x = RelDay, y= fGC)) +
  geom_line(data = Output, aes(x = x, y = y), size = 1) +
  geom_ribbon(data = Output, aes(x = x, ymin = lCI, ymax = UCI), alpha = 0.3) +   
  ylab("Fecal Glucocorticoids (ng/g)") + 
  xlab("Relative Days") + 
  ylim(20, 80) +
  theme_classic() + 
  theme(axis.title.y=element_text(family = "serif", size = 12),
        axis.title.x=element_text(family = "serif", size = 12),
        axis.text.y=element_text(family = "serif"),
        axis.text.x=element_text(family = "serif"),
        plot.title = element_text(hjust = -0.05, size = 12, family = "serif"))
```

```
#dev.off() # Activate if what to output to png
```

### Model 2: “Stress Response” Hypothesis

Our second model represents our hypothesis that stress levels may increase after animals are fitted with GPS collars and not decline. This is the worst case scenario. This model was represented by a piecewise regression model with a breakpoint, \(k\), and a change in intercept on the first day post-collaring \((k = 1)\). Here, this parameter (\(k\)) represents the time period it takes for stressors to be detected, based on the gut passage time of the animal. The parameter (k) could be estimated, but we fixed \(k\) to 1. The model is formulated as:

\[y\_i = \begin{cases}
\beta\_0 + \varepsilon\_i, & \text{for } t\_i < k \\
\beta\_0 + \beta\_1 + \varepsilon\_i, & \text{for } t\_i \ge k
\end{cases}
\]

\[\varepsilon\_i \sim Normal(0,\sigma^2)
\] where \(\beta\_0\) represents the mean initial level, \(\beta\_1\) represents the change in mean hormone levels beginning one day post-collaring, and \(t\_i\) represents the date of each observation relative to collaring. Note that the prior for \(\beta\_1\) allows FGM levels to decrease post-collaring, even though our expectation is that FGM levels will increase.

We save this model as `Model2_Stress.R` so that it can be sourced in **JAGS**. We expect this model to look similar to:


The model parameterization is:

```
model {
    
    # Priors
    b0 ~ dunif(0,100)
    b1 ~ dunif(-50,50) # Conceivable that stress went down (naive prediction)
    sd ~ dunif(0,25)
    tau <- 1/pow(sd,2)
    k <- 1

    # Likelihood
    for (i in 1:nobs) {
      y[i] ~ dnorm(lp[i], tau)
      lp[i] <- b0 + b1 * step(time[i] - k) # is the time before or after the breakpoint.  If after (t >= k), apply the change in intercept
    }
}
```

#### Model 2: Fit

Create a data list, define the parameters to report, source the model, and print model output.

```
# Create Data List
data <- list(
  nobs = nrow(collar),
  y = collar$fGC,
  time = collar$RelDay
)

# Define parameters
params <- c('b0','b1','sd','lp')

# Execute jags model
mod2 <- jags(data = data, inits = NULL, parameters.to.save = params, codaOnly = 'lp', model.file = "Model2_Stress.R", n.chains = 3, n.iter = n.iter, n.burnin = n.burnin, n.thin = n.thin, parallel=T)
```

```
## 
## Processing function input....... 
## 
## Done. 
##  
## Beginning parallel processing using 3 cores. Console output will be suppressed.
## 
## Parallel processing completed.
## 
## Calculating statistics.......
```

```
## Warning in doTryCatch(return(expr), name, parentenv, handler): At least one
## Rhat value could not be calculated.
```

```
## 
## Done.
```

```
print(mod2,3)
```

```
## JAGS output for model 'Model2_Stress.R', generated by jagsUI.
## Estimates based on 3 chains of 2e+05 iterations,
## adaptation = 100 iterations (sufficient),
## burn-in = 40000 iterations and thin rate = 100,
## yielding 4800 total samples from the joint posterior. 
## MCMC ran in parallel for 0.336 minutes at time 2019-12-18 21:12:43.
## 
##             mean    sd    2.5%     50%   97.5% overlap0     f  Rhat n.eff
## b0        45.867 1.910  42.084  45.862  49.586    FALSE 1.000 1.002   845
## b1        -3.121 2.608  -8.196  -3.136   2.006     TRUE 0.886 1.001  2199
## sd         9.467 0.951   7.837   9.381  11.497    FALSE 1.000 1.000  4800
## deviance 401.998 2.603 399.079 401.291 408.925    FALSE 1.000 1.000  4559
## 
## Successful convergence based on Rhat values (all < 1.1). 
## Rhat is the potential scale reduction factor (at convergence, Rhat=1). 
## For each parameter, n.eff is a crude measure of effective sample size. 
## 
## overlap0 checks if 0 falls in the parameter's 95% credible interval.
## f is the proportion of the posterior with the same sign as the mean;
## i.e., our confidence that the parameter is positive or negative.
## 
## DIC info: (pD = var(deviance)/2) 
## pD = 3.4 and DIC = 405.387 
## DIC is an estimate of expected predictive error (lower is better).
```

#### Model 2: Summarize

Summarize/view results. Calculate the highest posterior density intervals.

```
# Look at trace and density plots to assess model convergence
MCMCtrace(mod2, params = c('b0', 'b1'), ind=TRUE, pdf=FALSE)
```

```
# Summarize results.  Include median
Post.Summary <- MCMCsummary(mod2, 
                            params = c('b0', 'b1'),
                            Rhat = TRUE,
                            n.eff = TRUE,
                            func = function(x) c(median(x),hdi(x,credMass = 0.95)),
                            func_name = c('median','hdi_low','hdi_high'))

# View result summary
Post.Summary
```

```
##         mean       sd      2.5%       50%     97.5% Rhat n.eff    median
## b0 45.866933 1.909624 42.083947 45.862291 49.586017    1  4959 45.862291
## b1 -3.121424 2.607811 -8.195938 -3.136476  2.006417    1  4669 -3.136476
##      hdi_low  hdi_high
## b0 42.017414 49.492533
## b1 -8.188687  2.017038
```

```
# Export file
write.csv(Post.Summary, "./Output/Control/Model_Stress.csv")

# Calculate WAIC as in previous models
```

#### Model 2: Plot

Plot results.

```
# Setup x|y axes
Time <- seq(-8,10, by=0.01)
BPInd <- (Time >= 1)

Vpred <- mod2$mean$b0 + mod2$mean$b1*BPInd

# Create null objects to hold results
lCI <- UCI <- numeric()

for (i in 1:length(Time)){
  lCI[i] <- quantile(mod2$sims.list$b0 + mod2$sims.list$b1 * BPInd[i], probs=c(0.025))
  UCI[i] <- quantile(mod2$sims.list$b0 + mod2$sims.list$b1 * BPInd[i], probs=c(0.975))
}

# Create dataframe to plot in GGPlot
# Credible Intervals
Output.CI <- data.frame(x = Time,
                        lCI = lCI,
                        UCI = UCI)
# Pre-Treatment
Time1 <- Time[Time <1]
Vpred1 <- Vpred[Time <1]

Output.1 <- data.frame(x = Time1,
                       y = Vpred1)

# Post-Treatment
Time2 <- Time[Time>=1]
Vpred2 <- Vpred[Time>=1]

Output.2 <- data.frame(x = Time2,
                       y = Vpred2)

#png("./Output/Stress.png")
ggplot(collar) +
  geom_point(aes(x = RelDay, y= fGC)) +
  geom_line(data = Output.1, aes(x = x, y = y), size = 1) +
  geom_line(data = Output.2, aes(x = x, y = y), size = 1) +
  geom_ribbon(data = Output.CI, aes(x = x, ymin = lCI, ymax = UCI), alpha = 0.3) +   
  geom_vline(xintercept=1, colour = "gray", linetype = 3) + 
  ylab("Fecal Glucocorticoids (ng/g)") + 
  xlab("Relative Days") + 
  ylim(20, 80) +
  theme_classic() + 
  theme(axis.title.y=element_text(family = "serif", size = 12),
        axis.title.x=element_text(family = "serif", size = 12),
        axis.text.y=element_text(family = "serif"),
        axis.text.x=element_text(family = "serif"),
        plot.title = element_text(hjust = -0.05, size = 12, family = "serif"))
```

```
#dev.off()
```

### Model 3: “Habituation Response” Hypothesis

Our third model represents our hypothesis that stress levels will initially increase and gradually decline after animals are fitted with GPS collars, with animals becoming acclimated or habituated to the device. This model is formulated as:

\[y\_i = \begin{cases}
\beta\_0 + \varepsilon\_i, & \text{for } t\_i < k \\
\beta\_0 + \beta\_1 + \beta\_2(t\_i - k) + \varepsilon\_i, & \text{for } t\_i \ge k
\end{cases}
\]

\[\varepsilon\_i \sim Normal(0,\sigma^2)
\]

This includes two different intercepts (\(\beta\_0\) and \(\beta\_1\)), a breakpoint (\(k\)), and a slope parameter (\(\beta\_2\)). Here again, we have fixed the parameter \(k\) to 1. The slope parameter reflects our assumption that effects related to the collar should change slowly over time before returning to pre-treatment levels.

We save this model as `Model3_HabituationResponse.R` so that it can be sourced in **JAGS**. We expect this model to look similar to:


```
model {
    
    # Priors
    b0 ~ dunif(0,100)
    b1 ~ dunif(-50,50) # Conceivable that stress went down (naive prediction)
    b2 ~ dunif(-10,10)
    k1 <- 1 # Hard-coding based on biology
    sd ~ dunif(0,25)
    tau <- 1/pow(sd,2)

    # Likelihood
    for (i in 1:nobs) {
      y[i] ~ dnorm(lp[i], tau)
      lp[i] <- b0 + b1 * step(time[i] - k1) + b2*(time[i] - k1)*step(time[i] - k1) # is the time before or after the breakpoint.  If after (t >=k), apply the change in intercept
      }
}
```

#### Model 3: Fit

Create a data list, define the parameters to report, source the model, and print model output.

```
# Create Data List
data <- list(
  nobs = nrow(collar),
  y = collar$fGC,
  time = collar$RelDay
)

# Define parameters
params <- c('b0','b1','b2','sd','lp')

# Execute jags model
mod4 <- jags(data = data, inits = NULL, parameters.to.save = params, codaOnly = 'lp', 
             model.file = "Model3_HabituationResponse.R", n.chains = 3, n.iter = n.iter, n.burnin = n.burnin, n.thin = n.thin, parallel=T)
```

```
## 
## Processing function input....... 
## 
## Done. 
##  
## Beginning parallel processing using 3 cores. Console output will be suppressed.
## 
## Parallel processing completed.
## 
## Calculating statistics.......
```

```
## Warning in doTryCatch(return(expr), name, parentenv, handler): At least one
## Rhat value could not be calculated.
```

```
## 
## Done.
```

```
print(mod4,3)
```

```
## JAGS output for model 'Model3_HabituationResponse.R', generated by jagsUI.
## Estimates based on 3 chains of 2e+05 iterations,
## adaptation = 100 iterations (sufficient),
## burn-in = 40000 iterations and thin rate = 100,
## yielding 4800 total samples from the joint posterior. 
## MCMC ran in parallel for 0.489 minutes at time 2019-12-18 21:13:06.
## 
##             mean    sd    2.5%     50%   97.5% overlap0     f  Rhat n.eff
## b0        45.866 1.843  42.269  45.865  49.608    FALSE 1.000 1.000  4800
## b1         2.824 4.140  -5.338   2.717  10.995     TRUE 0.759 1.000  4800
## b2        -1.058 0.588  -2.187  -1.055   0.118     TRUE 0.962 1.001  3917
## sd         9.282 0.921   7.676   9.228  11.330    FALSE 1.000 1.000  4800
## deviance 399.597 2.921 395.898 398.956 406.966    FALSE 1.000 1.001  1752
## 
## Successful convergence based on Rhat values (all < 1.1). 
## Rhat is the potential scale reduction factor (at convergence, Rhat=1). 
## For each parameter, n.eff is a crude measure of effective sample size. 
## 
## overlap0 checks if 0 falls in the parameter's 95% credible interval.
## f is the proportion of the posterior with the same sign as the mean;
## i.e., our confidence that the parameter is positive or negative.
## 
## DIC info: (pD = var(deviance)/2) 
## pD = 4.3 and DIC = 403.86 
## DIC is an estimate of expected predictive error (lower is better).
```

#### Model 3: Summarize

Summarize/view results. Calculate the highest posterior density intervals.

```
# Look at trace and density plots to assess model convergence
MCMCtrace(mod4, params = c('b0', 'b1', 'b2'), ind=TRUE, pdf=FALSE)
```

```
# Summarize results.  Include median
Post.Summary <- MCMCsummary(mod4, 
                            params = c('b0', 'b1', 'b2'),
                            Rhat = TRUE,
                            n.eff = TRUE,
                            func = function(x) c(median(x),hdi(x,credMass = 0.95)),
                            func_name = c('median','hdi_low','hdi_high'))

# View result summary
Post.Summary
```

```
##         mean        sd      2.5%       50%      97.5% Rhat n.eff    median
## b0 45.865534 1.8426733 42.269125 45.865217 49.6081739    1  4800 45.865217
## b1  2.823946 4.1396160 -5.338425  2.717160 10.9946702    1  4800  2.717160
## b2 -1.058441 0.5882506 -2.186947 -1.055446  0.1175913    1  5407 -1.055446
##      hdi_low    hdi_high
## b0 41.860106 49.17583411
## b1 -5.220178 11.07245938
## b2 -2.213923  0.07929267
```

```
# Export file
write.csv(Post.Summary, "./Output/Model_Habituation.csv")

# Calculate WAIC as in previous models
```

#### Model 3: Plot

Plot results

```
Time <- seq(-8,10, by=0.1)
BPInd <- (Time >= 1)

Vpred <- mod4$mean$b0 + mod4$mean$b1 * BPInd + mod4$mean$b2 * BPInd * (Time - 1)

lCI <- UCI <- numeric()

for (i in 1:length(Time)){
  lCI[i] <- quantile(mod4$sims.list$b0 + mod4$sims.list$b1 * BPInd[i] + mod4$sims.list$b2 * BPInd[i] * (Time[i] - 1), probs=c(0.025))
  UCI[i] <- quantile(mod4$sims.list$b0 + mod4$sims.list$b1 * BPInd[i] + mod4$sims.list$b2 * BPInd[i] * (Time[i] - 1), probs=c(0.975))
}

Output.CI <- data.frame(x = Time,
                        lCI = lCI,
                        UCI = UCI)
# Pre-treatment
Time1 <- Time[Time < 1]
Vpred1 <- Vpred[Time < 1]

Output.1 <- data.frame(x = Time1,
                       y = Vpred1)

# Post-treatment
Time2 <- Time[Time >= 1]
Vpred2 <- Vpred[Time >= 1]

Output.2 <- data.frame(x = Time2,
                       y = Vpred2)

#png(paste0("./Output/Habituation.png")
ggplot(collar) +
  geom_point(aes(x = RelDay, y= fGC)) +
  geom_line(data = Output.1, aes(x = x, y = y), size = 1) +
  geom_line(data = Output.2, aes(x = x, y = y), size = 1) +
  geom_ribbon(data = Output.CI, aes(x = x, ymin = lCI, ymax = UCI), alpha = 0.3) +
  geom_vline(xintercept=1, colour = "gray", linetype = 3) + 
  ylab("Fecal Glucocorticoids (ng/g)") + 
  xlab("Relative Days") + 
  ylim(20, 80) +
  theme_classic() + 
  theme(axis.title.y=element_text(family = "serif", size = 12),
        axis.title.x=element_text(family = "serif", size = 12),
        axis.text.y=element_text(family = "serif"),
        axis.text.x=element_text(family = "serif"),
        plot.title = element_text(hjust = -0.05, size = 12, family = "serif"))
```

```
#dev.off()
```

### Model 4: “Handling Response” Hypothesis

Our fourth model represents our hypothesis that stress levels may change after animals are fitted with GPS collars, temporarily increasing (or decreasing), before returning to pre-treatment levels. This model is formulated as:

\[y\_i = \begin{cases}
\beta\_0 + \varepsilon\_i, & \text{for } t\_i < k\_1 \\
\beta\_0 + \beta\_1 + \varepsilon\_i, & \text{for } k\_1 \le t\_i < k\_2 \\
\beta\_0 + \varepsilon\_i, & \text{for } t\_i \ge k\_2
\end{cases}
\]

\[\varepsilon\_i \sim Normal(0,\sigma^2)
\] where \(\beta\_0\) represents the mean initial FGM level and \(\beta\_1\) represents the change in hormone levels between breakpoints \(k\_1\) and \(k\_2\). As such, this model includes an intercept (\(\beta\_0\)), a treatment effect (\(\beta\_1\)), and two different breakpoints (\(k\_1\) and \(k\_2\)). We estimate the second breakpoint from the data (\(k\_2\)), but set \(k\_1\) to 1.

We save this model as `Model4_HandlingResponse.R` so that it can be sourced in **JAGS**. We expect this model to look similar to:


The model parameterization is:

```
model {
    
    # Priors
    b0 ~ dunif(0,100)
    b1 ~ dunif(-50,50) # Conceivable that stress went down (naive prediction)
    sd ~ dunif(0,25)
    tau <- 1/pow(sd,2)
    k1 <- 1
    k2 ~ dunif(1.001,9.999) # Return to normal needs to be between days 2 and 10 (can't be within first day
    
    # Likelihood
    for (i in 1:nobs) {
      y[i] ~ dnorm(lp[i], tau)
      lp[i] <- b0 + b1 * step(time[i] - k1) * (1 - step(time[i] - k2))
      }
}
```

#### Model 4: Fit

Create a data list, define the parameters to report, source the model, and print model output.

```
# Create Data List
data <- list(
  nobs = nrow(collar),
  y = collar$fGC,
  time = collar$RelDay
)

# Define parameters
params <- c('b0','b1','k2','sd','lp')

# Execute jags model
mod3 <- jags(data = data, inits = NULL, parameters.to.save = params, codaOnly = 'lp', model.file = "Model4_HandlingResponse.R", n.chains = 3, n.iter = n.iter, n.burnin = n.burnin, n.thin = n.thin, parallel=T)
```

```
## 
## Processing function input....... 
## 
## Done. 
##  
## Beginning parallel processing using 3 cores. Console output will be suppressed.
## 
## Parallel processing completed.
## 
## Calculating statistics.......
```

```
## Warning in doTryCatch(return(expr), name, parentenv, handler): At least one
## Rhat value could not be calculated.
```

```
## 
## Done.
```

```
print(mod3,3)
```

```
## JAGS output for model 'Model4_HandlingResponse.R', generated by jagsUI.
## Estimates based on 3 chains of 2e+05 iterations,
## adaptation = 100 iterations (sufficient),
## burn-in = 40000 iterations and thin rate = 100,
## yielding 4800 total samples from the joint posterior. 
## MCMC ran in parallel for 0.427 minutes at time 2019-12-18 21:13:37.
## 
##             mean    sd    2.5%     50%   97.5% overlap0     f  Rhat n.eff
## b0        43.323 1.433  40.624  43.264  46.305    FALSE 1.000 1.000  4210
## b1         6.646 5.705  -6.261   7.339  16.087     TRUE 0.885 1.001  4800
## k2         3.993 1.544   1.384   3.743   8.853    FALSE 1.000 1.000  4800
## sd         9.280 0.946   7.630   9.211  11.345    FALSE 1.000 1.000  4800
## deviance 399.383 3.584 394.902 398.467 407.768    FALSE 1.000 1.000  4800
## 
## Successful convergence based on Rhat values (all < 1.1). 
## Rhat is the potential scale reduction factor (at convergence, Rhat=1). 
## For each parameter, n.eff is a crude measure of effective sample size. 
## 
## overlap0 checks if 0 falls in the parameter's 95% credible interval.
## f is the proportion of the posterior with the same sign as the mean;
## i.e., our confidence that the parameter is positive or negative.
## 
## DIC info: (pD = var(deviance)/2) 
## pD = 6.4 and DIC = 405.806 
## DIC is an estimate of expected predictive error (lower is better).
```

#### Model 4: Summarize

Summarize/view results. Calculate the highest posterior density intervals.

```
# Look at trace and density plots to assess model convergence
MCMCtrace(mod3, params = c('b0', 'b1', 'k2'), ind=TRUE, pdf=FALSE)
```

```
# Summarize results.  Include median
Post.Summary <- MCMCsummary(mod3, 
                            params = c('b0', 'b1', 'k2'),
                            Rhat = TRUE,
                            n.eff = TRUE,
                            func = function(x) c(median(x),hdi(x,credMass = 0.95)),
                            func_name = c('median','hdi_low','hdi_high'))

# View result summary
Post.Summary
```

```
##         mean       sd      2.5%       50%     97.5% Rhat n.eff    median
## b0 43.322966 1.432850 40.624191 43.263567 46.304844    1  4800 43.263567
## b1  6.646409 5.704742 -6.261395  7.338879 16.086693    1  5210  7.338879
## k2  3.993471 1.543674  1.384341  3.742979  8.853059    1  5128  3.742979
##      hdi_low  hdi_high
## b0 40.742081 46.397290
## b1 -5.052812 16.875247
## k2  1.001205  7.676337
```

```
# Export file
write.csv(Post.Summary, "./Output/Model_Handling.csv")

# Calculate WAIC as in previous models
```

#### Model 4: Plot

Plot results

```
Time <- seq(-8,10, by=0.1)
BPInd <- (Time >= 1)
BPInd2 <- (Time < (ceiling(mod3$mean$k2)))

Vpred <- mod3$mean$b0 + mod3$mean$b1*BPInd * BPInd2

lCI <- UCI <- numeric()

for (i in 1:length(Time)){
  lCI[i] <- quantile(mod3$sims.list$b0 + mod3$sims.list$b1 * BPInd[i] * (Time[i] < ceiling(mod3$sims.list$k2)), probs=c(0.025))
  UCI[i] <- quantile(mod3$sims.list$b0 + mod3$sims.list$b1 * BPInd[i] * (Time[i] < ceiling(mod3$sims.list$k2)), probs=c(0.975))
}

# Create dataframe to plot in GGPlot
Output.CI <- data.frame(x = Time,
                        lCI = lCI,
                        UCI = UCI)

# Pre-Treatment
Time1 <- Time[Time <1]
Vpred1 <- Vpred[Time <1]

Output.1 <- data.frame(x = Time1,
                       y = Vpred1)

# Treatment
Time2 <- Time[Time >=1 & Time < ceiling(mod3$mean$k2)]
Vpred2 <- Vpred[Time >=1 & Time < ceiling(mod3$mean$k2)]

Output.2 <- data.frame(x = Time2,
                       y = Vpred2)

# Post-Treatment
Time3 <- Time[Time >= ceiling(mod3$mean$k2)]
Vpred3 <- Vpred[Time >= ceiling(mod3$mean$k2)]

Output.3 <- data.frame(x = Time3,
                       y = Vpred3)

#png("./Output/Handling.png")
ggplot(collar) +
  geom_point(aes(x = RelDay, y= fGC)) +
  geom_line(data = Output.1, aes(x = x, y = y), size = 1) +
  geom_line(data = Output.2, aes(x = x, y = y), size = 1) +
  geom_line(data = Output.3, aes(x = x, y = y), size = 1) +
  geom_ribbon(data = Output.CI, aes(x = x, ymin = lCI, ymax = UCI), alpha = 0.3) + 
  geom_vline(xintercept=1, colour = "gray", linetype = 3) + 
  ylab("Fecal Glucocorticoids (ng/g)") + 
  xlab("Relative Days") + 
  ylim(20, 80) +
  theme_classic() + 
  theme(axis.title.y=element_text(family = "serif", size = 12),
        axis.title.x=element_text(family = "serif", size = 12),
        axis.text.y=element_text(family = "serif"),
        axis.text.x=element_text(family = "serif"),
        plot.title = element_text(hjust = -0.05, size = 12, family = "serif"))
```

```
#dev.off()
```

### Leave-One-Out Cross-Validation

Lastly, we describe how to programatically code the leave-one-out cross-validation. We illustrate this with our simplest model (simple linear response with no slope), but the code could be easily adapted to include all four models. A leave-one-out approach repeatedly refits the model, withholding a single record each time during model fitting to use as validation. We then sum the squared errors to provide an overall evaluation of the fit of the model.

```
# Create a matrix to hold residual error after each step
errors <- matrix(nrow=dim(collar)[1], ncol = 1)
# This could be updated (ncol = 4) to include errors calculated for the other models
# The errors could then be iteratively saved in the matrix

# Loop through every row in the dataset
for (sample in 1:nrow(collar)){
    print(paste0("Sample ",sample," of ",nrow(collar)))
  
    # Separate the test and training dataset during each step
    test <- collar[sample,]  
    train <- collar[-sample,]

    # Create Data List
    data <- list(
      nobs = nrow(train),
      y = train$fGC
    )
    
    # Define parameters
    params <- c('b0','sd')
    
    # Execute jags model
    mod1 <- jags(data = data, inits = NULL, parameters.to.save = params, 
                 model.file = "Model1_Linear.R", n.chains = 3, n.iter = n.iter, n.burnin = n.burnin, n.thin = n.thin, parallel=T)
    
    # Print model summary
    #print(mod1,3)
    
    # Calculate residual error
    # Subtract the fGC level in the test dataset (the withheld data) with the model prediction
    # Save the errors to the errors matrix
    errors[sample,1] <- test$fGC - mod1$mean$b0
    
    # Calculating the residual error is the only difficult part of the code
    # It entails understanding the model statement and appropriately coding it.
    # For our models, after they were executed in turn, would be coded:
    # Model 2:
        #errors[sample,2] <- test$fGC - (mod2$mean$b0 + mod2$mean$b1 * (test$RelDay >= 1))
    # Model 3:
        #errors[sample,3] <- test$fGC - (mod3$mean$b0 + mod3$mean$b1 * ((test$RelDay >= 1) & (test$RelDay < mod3$mean$k2)))
    # Model 4:
        #errors[sample,4] <- test$fGC - (mod4$mean$b0 + mod4$mean$b1 * (test$RelDay >= 1) + mod4$mean$b2 * (test$RelDay - 1) * (test$RelDay >= 1))
}
```

```
# Summarize and Display errors to provide an evaluation of model fit
# This value then can be compared to the SSE from the other models
(SSE <- colSums(errors^2))
```

```
## [1] 4843.14
```
