# Supplementary material for: Short-term effects of GPS collars on the activity, behavior, and adrenal response of scimitar-horned oryx (Oryx dammah)
Source: PLoS One. 2020 Feb 11;15(2):e0221843. doi: 10.1371/journal.pone.0221843 (PMC7012457; doi:10.1371/journal.pone.0221843)
Supplement: S3 Code — Script and data tutorial to quantify the short-term decline in headshaking that occurred after scimitar-horned oryx (Oryx dammah) were fitted with GPS collars. Headshaking, captured in data collected from tri-axial accelerometers, was classified by random forest models with a high degree of accuracy and precision. Data were then aggregated to an hourly time window. Competing time-series models were then used to calculate the amount of headshaking that occurred over time. (ZIP) [file pone.0221843.s003.zip › S3_Code/AccelerometerAnalysis_NegativeExponentialDecay.html]

Time Series Analysis: Accelerometer Data


# Time Series Analysis: Accelerometer Data

### *Stabach et al. 2019 - Effects of GPS Collars*

#### *Jared Stabach, Smithsonian Conservation Biology Institute*

#### *2019-December-18*

# Binomial Response Modeling

> Comments/Questions: Contact Grant Connette (connetteg@si.edu) and Jared Stabach (stabachj@si.edu)

Here we illustrate time series analyses applied to quantify the short-term decline in headshaking that occurred after scimitar-horned oryx were fitted with GPS collars. Headshaking, captured in data collected from tri-axial accelerometers, was classified by random forest models with a high degree of accuracy and precision (see Stabach et al. 2019). Data were then aggregated to an hourly time window (data shown in black dots in the figure below). Competing time-series models include:

1. Binomial response model with a negative exponential decay (red dotted line);
2. Binomial response model with a negative exponential decay and a day/night switch (blue dashed line); and
3. Binomial response model with a negative exponential decay and a series of harmonics to fit the day/night fluctuations (green solid line).

In the example below, we illustrate results only for ‘114839’ (Chari), although the dataset for all individuals is provided and models could be easily applied to data for each individual. All models are fit in a Bayesian framework. We also provide instruction and code for evaluating model support using Leave-One-Out cross-validation. By following this example, you will be able to recreate the following figure:


Please see Stabach et al. 2019 for additional details:

Stabach, J.A., S.A. Cunningham, G. Connette, J.L. Mota, D. Reed, M. Byron, M. Songer, T. Wacher, K. Mertes, J.L. Brown, P. Comizzoli, J. Newby, S. Monfort, and P. Leimgruber. In Review. Short-term effects of GPS collars on behavior and stress in scimitar-horned oryx (*Oryx dammah*). PlosOne.

### Load Libraries

Load each library necessary to complete the analysis.

```
# Clear objects in memory
rm(list=ls())

# Load library
library(jagsUI)
library(ggplot2)
library(MCMCvis)
library(HDInterval)
library(scales)
```

### Read/Prepare Data

The data, which consist of counts of headshaking, have been aggregated to an hourly time window. We read the data into R, calculate the total number of observations at each hour, and re-define the time as the number of hours since GPS collars were fitted on animals. Thus, the time value is a relative time. The accelerometer takes positions at 8 positiosn per second (8 Hz). The number of measurements collected in one hour is approximately 28800 (60 seconds per minute \* 60 minutes per hour \* 8 position per second)

```
# Read the dataset
dat_all <- read.csv("./Data/Activity_AllAnimals2.csv")

# Calculate total number of binomial trials for each observation
dat_all$K <- rowSums(dat_all[4:7])

# Make first hour equal to time 0, not 1
dat_all$X <- dat_all$X - 1

# Look at data
head(dat_all)
```

```
##   X      Date Hour  FEED HDSHK  LOCO  REST Animal     K
## 1 0 11/2/2015   11  2755   738 24819   485  Chari 28797
## 2 1 11/2/2015   12  8192  1044 16376  3190  Chari 28802
## 3 2 11/2/2015   13 10319  1206  8785  8490  Chari 28800
## 4 3 11/2/2015   14 10040  1107  7611 10032  Chari 28790
## 5 4 11/2/2015   15  5630   557  5827 16786  Chari 28800
## 6 5 11/2/2015   16  7857   160  2855 17930  Chari 28802
```

### Data Analysis

You’ll notice that beyond ‘headshaking’, our dataset includes behaviors such as ‘Feeding’, ‘Locomotion’, and ‘Resting’. This means that our analysis could be adapted to any one of these behaviors.

We fit the model in a Bayesian framework using Markov chain Monte Carlo (MCMC) simulation. To initiate the analysis, we first setup the number of iterations, burn-in, and thinning rate. Note, the number of iterations (`n.iter`) has been reduced in the code (i.e., set to 200,000) so that analyses will execute quickly. `n.iter` should be increased in subsequent analyses to make sure the entirety of the parameter space has been thoroughly explored.

For results presented in our manuscript, we ran three parallel Markov chain Monte Carlo (MCMC) chains for 400,000 iterations, discarding the first 80,000 iterations of each chain as burn-in, and thinning the remaining posterior samples at a rate of 1:100. Thus, our conclusions are based on 9,600 samples from the joint posterior distribution. Convergence was assessed by visual inspection of traceplots to ensure a reasonable exploration of the parameter space and by ensuring that the potential scale reduction factor was < 1.1 for each parameter (Gelman & Rubin 1992).

```
# Set-up burn-in/iterations for JAGS}
n.iter <- 200000
n.burnin <- n.iter*0.20
n.thin <- 100
```

### Data Subset

Models were fit individually to accelerometer datasets for each animal. This means that we must first subset the dataframe to each animal. We also truncate the dataset to remove observations taken after an animal subsequently handled. We will only fit the first animal in our dataset as an example here, but do provide ‘commented’ code so that the ‘for loop’ can be activated, to analyze data for all individuals.

```
# Extract unique animal names
# This will be used to 'loop' the analysis over every animal in the dataframe
animals <- unique(dat_all$Animal)

# The four animals included in analysis are appended sequentially in the dataframe
# Since animals were handled on multiple occassions, we truncate each animals' results to remove subsequent handling events
# This is unique to this dataset
NextHandle <- c(37,227,225,231)

# Create an output directory
Output.dir <- "./Output/"

if (!dir.exists(Output.dir)){
  dir.create(Output.dir)
} else {
  print("Directory already exists!")
}
```

```
## [1] "Directory already exists!"
```

```
# Legend Labels
# Simply items used for graphing at the end of the code
Llabel1 <- c(0.04,0.07,0.04,0.07)
Llabel2 <- c(10,125,150,125)

# Determine the animal to analyze
# Here, we analyze animal No. 1 in our data list
# However, this could be placed a loop so that models for each animal are fit

# Specify individual
id <- 1
# Or activate the loop
#for (id in 1:4){
  # Print the name of the animal included
  print(paste0("Starting Animal: ",animals[id]))
```

```
## [1] "Starting Animal: Chari"
```

```
  # Subset dataset
  dat <- subset(dat_all, Animal==animals[id])
  # Further subset dataset to remove the records before the secondary handling event. 
  # Use 'NextHandle' object created above
  dat <- subset(dat, X<=NextHandle[id])
```

### Model 1: Negative Exponential Decay

Our first model assumes the collaring effect gradually wears off after increasing during the time period immediately after being fitted with a GPS collar.

\[y\_t \sim Binomial(p\_t, N\_t)\] \[logit(p\_t) = ae^{-bt} + c\]

Here, \(a\) represents the initial change in headshaking due to collaring, \(b\) represents the decay rate, and \(c\) is an asymptote representing the baseline level of headshaking in the absence of a collaring effect.

We assigned diffuse \(Uniform(0,5)\) and \(Uniform(-10,10)\) priors to represent post-collaring increases (\(a\)) and baseline logit-scale headshaking probability (\(c\)), respectively. The exponential decay rate, \(b\), was assigned a diffuse \(Uniform(-1,1)\) prior. We also calculate the halflife (\(log(2/b)\)) of the treatment effect, which represents the time required for the treatment effect to decline to half its initial magnitude.

We save our model parameterization as `1_NegExp_Binom.R`.

The model parameterization is:

```
model{
  
  # Priors
  a ~ dunif(0,5) # could be -5,5, check with plogis(-5) to make sure covering proper range
  b ~ dunif(-1,1)
  c ~ dunif(-10,10)
  
  for (i in 1:nobs){
    y[i] ~ dbinom(p[i],K[i])
    logit(p[i]) <- a * exp(-b*time[i]) + c
    }
  
  # Derived Quantities
  halflife <- log(2/b)
}
```

#### Model 1: Fit

We create a data list, define the parameters we want reported as output from the model, run the model in **JAGS**, and summarize results.

```
# Create Data List
data <- list(
  nobs = nrow(dat), #Total number of hourly measurements analyzed
  y = dat$HDSHK, #Counts of head-shaking events (hourly)
  time = dat$X, #Hours since collaring
  K = dat$K #Total number of accelerometer measurements each hour
)

# Create initial/starting values
inits <- function(){
  list(
    a = 2,
    b = 0.01,
    c = -3
  )
}

# Define parameters
params <- c('a','b','c','halflife','p')

# Execute jags model
mod1 <- jags(data = data, inits = inits, parameters.to.save = params, codaOnly = c("p"), 
             model.file = "1_NegExp_Binom.R", n.chains = 3, n.iter = n.iter, n.burnin = n.burnin, n.thin = n.thin, parallel=T)
```

```
## 
## Processing function input....... 
## 
## Done. 
##  
## Beginning parallel processing using 3 cores. Console output will be suppressed.
## 
## Parallel processing completed.
## 
## Calculating statistics....... 
## 
## Done.
```

```
print(mod1,3)
```

```
## JAGS output for model '1_NegExp_Binom.R', generated by jagsUI.
## Estimates based on 3 chains of 2e+05 iterations,
## adaptation = 100 iterations (sufficient),
## burn-in = 40000 iterations and thin rate = 100,
## yielding 4800 total samples from the joint posterior. 
## MCMC ran in parallel for 0.5 minutes at time 2019-12-18 20:32:02.
## 
##              mean    sd     2.5%      50%    97.5% overlap0 f Rhat n.eff
## a           2.385 0.028    2.331    2.385    2.440    FALSE 1    1  4800
## b           0.208 0.005    0.199    0.208    0.217    FALSE 1    1  4052
## c          -5.379 0.017   -5.412   -5.379   -5.346    FALSE 1    1  3590
## halflife    2.263 0.022    2.221    2.263    2.308    FALSE 1    1  4098
## deviance 9869.739 2.524 9866.943 9869.069 9876.325    FALSE 1    1  4800
## 
## Successful convergence based on Rhat values (all < 1.1). 
## Rhat is the potential scale reduction factor (at convergence, Rhat=1). 
## For each parameter, n.eff is a crude measure of effective sample size. 
## 
## overlap0 checks if 0 falls in the parameter's 95% credible interval.
## f is the proportion of the posterior with the same sign as the mean;
## i.e., our confidence that the parameter is positive or negative.
## 
## DIC info: (pD = var(deviance)/2) 
## pD = 3.2 and DIC = 9872.926 
## DIC is an estimate of expected predictive error (lower is better).
```

#### Model 1: Summarize and Plot

Calculate the highest posterior density intervals and create trace plots

```
# Look at trace and density plots to assess model convergence
MCMCtrace(mod1, ind=TRUE, params = c('a','b','c','halflife'), pdf=FALSE)
```

```
# Summarize results.  Include median
Post.Summary1 <- MCMCsummary(mod1, 
                             params = c('a','b','c','halflife'),
                             Rhat = TRUE,
                             n.eff = TRUE,
                             func = function(x) c(median(x),hdi(x,credMass = 0.95)),
                             func_name = c('median','hdi_low','hdi_high'))

# View result summary if want to see results
#Post.Summary1

# Plot results
par(mfrow=c(1,1))
plot(dat$X,dat$HDSHK/dat$K,type="b",xlim=c(0,nrow(dat)),pch = 15,cex = 0.5, ylim=c(0,0.06), las = 1, xlab="Hours since collaring", ylab="Headshaking", bty="n")
lines(dat$X,plogis(mod1$mean$a * exp(-mod1$mean$b*dat$X) + mod1$mean$c),type="l",col="red",lty=3,lwd=1)

# Legend
legend(x = 20,y = 0.05, legend="Binomial [NExp Decay]",lty=5, lwd=1, col = "red", bty = "n")
```

#### Model 1: Calculate Metrics and Save

Calculate derived quantities from the results and save to the output directory

```
# Calculate:
# Background (recovery) rate
# Headshaking rate immediately after collaring
# Treatment effect (percent increase)
Recovered <- plogis(mod1$sims.list$c)
Handled <- plogis(mod1$sims.list$a + mod1$sims.list$c)
Mod1EffectSize <- (Handled-Recovered)/Recovered*100

# Create function to calculate mextrics on each derived quantity:
Calc.Stats <- function(x) c(mean = mean(x), sd = sd(x), median = median(x), quant25 = quantile(x, probs = 0.025), quant975 = quantile(x,probs = 0.975))

Recov.Stats <- Calc.Stats(Recovered)
Handle.Stats <- Calc.Stats(Handled) 
Mod1EffectSize.Stats <- Calc.Stats(Mod1EffectSize)

# Bind the results to Post.Summary1 for export
Post.Summary1 <- rbind(Post.Summary1,Recov.Stats,Handle.Stats,Mod1EffectSize.Stats)

# Write file
write.csv(Post.Summary1, paste0(Output.dir,"/Model1_Summary_",animals[id],".csv"))
```

### Model 2: Negative Exponential Decay with Day/Night Switch

Our second model is similar to our first and assumes that headshaking will decline over time based on an exponential decay process. The key difference between this model and our first model, is that we account for variation in the level of headshaking between daytime (08:00 to 16:00) periods when oryx are active, and nighttime periods (17:00 to 7:00) when oryx are largely inactive. We add a binary indicator variable (\(Day\_t\)) to indicate whether each count occured during the daytime (\(Day\_t = 1\)) or nighttime (\(Day\_t = 0\)). The parameter \(d\) is an exponent parameter that allows for relative amplification (or reduction) in the probability of headshaking during daytime hours. This model is formulated as:

\[y\_t \sim Binomial(p\_t, N\_t)\] \[p\_t = \begin{cases}
\varphi\_t, & \text{if } Day\_t = 0 \\
\varphi\_td, & \text{if } Day\_t = 1
\end{cases}\]

\[logit(\varphi\_t) = ae^{-bt}+c \] As with our previous model, we assigned diffuse \(Uniform(0,5)\) and \(Uniform(-10,10)\) priors to represent post-collaring increases (\(a\)) and baseline logit-scale headshaking probability (\(c\)), respectively. The exponential decay rate, \(b\), was assigned a diffuse \(Uniform(-1,1)\) prior. Parameter \(d\), which controls the relative increase in day time vs. night time headshaking levels, was assigned a diffuse \(Uniform(0,5)\) prior. We also calculated the halflife (\(log(2/b)\)) of the treatment effect, which represents the time required for the treatment effect to decline to half its initial magnitude.

We save our model parameterization as `2_NegExp_Hour_Binomial.R`.

```
model{
    
  # Priors
  a ~ dunif(0,5)
  b ~ dunif(-1,1)
  c ~ dunif(-10,10)
  d ~ dunif(0,5)
    
    for (i in 1:nobs){
      y[i] ~ dbinom(p[i],K[i])
      
      # Determine if a given hour is a Day or Night using the step function
      p[i] <- p_Night[i] * (1-step(hour[i]-8)*step(16-hour[i])) + pow((p_Night[i] * step(hour[i]-8)*step(16-hour[i])), d)
      logit(p_Night[i]) <- (a * exp(-b*time[i]) + c) 
      }   
    
    # Derived Quantities
    halflife <- log(2/b)
}
```

#### Model 2: Fit

We create a data list, define the parameters we want reported from the model, run the model in **JAGS**, and summarize results.

```
# Create Data List
data <- list(
  nobs = nrow(dat),
  y = dat$HDSHK,
  time = dat$X,
  K = dat$K,
  hour = dat$Hour
)

inits <- function(){
  list(
    a = 2,
    b = 0.01,
    c = -3,
    d = 1
  )
}

params <- c('a','b','c','d','halflife','p')

# Execute jags model
mod1.1 <- jags(data = data, inits = inits, parameters.to.save = params, codaOnly = c("p"), model.file = "2_NegExp_Hour_Binomial.R", n.chains = 3, n.iter = n.iter, n.burnin = n.burnin, n.thin = n.thin, parallel=T)
```

```
## 
## Processing function input....... 
## 
## Done. 
##  
## Beginning parallel processing using 3 cores. Console output will be suppressed.
## 
## Parallel processing completed.
## 
## Calculating statistics....... 
## 
## Done.
```

```
print(mod1.1,3)
```

```
## JAGS output for model '2_NegExp_Hour_Binomial.R', generated by jagsUI.
## Estimates based on 3 chains of 2e+05 iterations,
## adaptation = 100 iterations (sufficient),
## burn-in = 40000 iterations and thin rate = 100,
## yielding 4800 total samples from the joint posterior. 
## MCMC ran in parallel for 0.874 minutes at time 2019-12-18 20:32:34.
## 
##              mean    sd     2.5%      50%    97.5% overlap0 f  Rhat n.eff
## a           1.762 0.043    1.679    1.762    1.848    FALSE 1 1.001  2240
## b           0.176 0.009    0.158    0.176    0.194    FALSE 1 1.000  3277
## c          -6.999 0.042   -7.081   -6.999   -6.917    FALSE 1 1.000  4800
## d           0.622 0.004    0.614    0.622    0.630    FALSE 1 1.001  3885
## halflife    2.432 0.052    2.331    2.432    2.537    FALSE 1 1.000  3330
## deviance 3197.898 2.841 3194.411 3197.220 3205.362    FALSE 1 1.000  4800
## 
## Successful convergence based on Rhat values (all < 1.1). 
## Rhat is the potential scale reduction factor (at convergence, Rhat=1). 
## For each parameter, n.eff is a crude measure of effective sample size. 
## 
## overlap0 checks if 0 falls in the parameter's 95% credible interval.
## f is the proportion of the posterior with the same sign as the mean;
## i.e., our confidence that the parameter is positive or negative.
## 
## DIC info: (pD = var(deviance)/2) 
## pD = 4 and DIC = 3201.936 
## DIC is an estimate of expected predictive error (lower is better).
```

#### Model 2: Summarize and Plot

Calculate the highest posterior density intervals and create trace plots

```
# Look at trace and density plots to assess model convergence
MCMCtrace(mod1, ind=TRUE, params = c('a','b','c','d','halflife'), pdf=FALSE)
```

```
## Warning in MCMCchains(object, params, excl, ISB, mcmc.list = TRUE): "d" not
## found in MCMC output.
```

```
# Summarize results.  Include median
Post.Summary2 <- MCMCsummary(mod1.1, 
                             params = c('a','b','c','d','halflife'),
                             Rhat = TRUE,
                             n.eff = TRUE,
                             func = function(x) c(median(x),hdi(x,credMass = 0.95)),
                             func_name = c('median','hdi_low','hdi_high'))

# View result summary
#Post.Summary2

# Plot results, including results from model 1
par(mfrow=c(1,1))
plot(dat$X,dat$HDSHK/dat$K,type="b",xlim=c(0,nrow(dat)),pch = 15,cex = 0.5, ylim=c(0,0.06), las = 1, xlab="Hours since collaring", ylab="Headshaking", bty="n")
lines(dat$X,plogis(mod1$mean$a * exp(-mod1$mean$b*dat$X) + mod1$mean$c),type="l",col="red",lty=3,lwd=1)
lines(dat$X,(plogis(mod1.1$mean$a * exp(-mod1.1$mean$b*dat$X) + mod1.1$mean$c)*!(dat$Hour %in% 8:16)) +
      ((plogis(mod1.1$mean$a * exp(-mod1.1$mean$b*dat$X) + mod1.1$mean$c)*(dat$Hour %in% 8:16))^mod1.1$mean$d),
      type="l",col="blue",lty=2,cex=1.2,lwd=2) 

# Legend
legend(x = 15,y = 0.05, legend=c("Binomial [NExp Decay]","Binomial [NExp + Day/Night]"),lty=c(5,2), lwd=c(1,1), col = c("red","blue"), bty = "n")
```

#### Model 2: Calculate Metrics and Save

Calculate derived quantities from the results and save to the output directory

```
# Calculate:
# Background (recovery) rate for one 24-hour cycle
# Headshaking rate immediately after collaring
# Treatment effect (percent increase)
Recovered <- ((plogis(mod1.1$sims.list$c)^mod1.1$sims.list$d)*9 + plogis(mod1.1$sims.list$c)*15)/24
Handled <- ((plogis(mod1.1$sims.list$a + mod1.1$sims.list$c)^mod1.1$sims.list$d)*9 + plogis(mod1.1$sims.list$a + mod1.1$sims.list$c)*15)/24
Mod1.1EffectSize <- (Handled-Recovered)/Recovered*100

Recov.Stats <- Calc.Stats(Recovered)
Handle.Stats <- Calc.Stats(Handled)
Mod1.1EffectSize.Stats <- Calc.Stats(Mod1.1EffectSize)

# Bind the results to Post.Summary2 for export
Post.Summary2 <- rbind(Post.Summary2,Recov.Stats,Handle.Stats,Mod1.1EffectSize.Stats)

# Write file
write.csv(Post.Summary2, paste0(Output.dir,"/Model2_Hour_Summary_",animals[id],".csv"))
```

### Model 3: Negative Exponential Decay with Harmonics

Our final approach borrows pieces from our previous two models, but models the time series of hourly headshaking as a combination of two harmonimc processes. Here, the frequency of headshaking, \(y\_t\), was treated as a binomial response with a time-specific probability of headshaking, \(p\_t\), and known number of binomial trials, \(N\_t\). The probability of headshaking was then specified as a mixture of harmonic processes (e.g., Shumway & Stoffer 2017), with two complementary mixture weight parameters, \(\alpha\_{1t}\) and \(\alpha\_{2t}\), indicating the proportional contribution of each harmonic process, \(k\), to the logit-scale probability of headshaking at each time \(t\). The two harmonic processes were each composed of a process-specific mean, \(\mu k\), and a background series of daily oscillations, \(U\_{k1} cos(2\pi wt)\) + \(U\_{k2} sin(2\pi wt)\), where \(U\_{k1}\) and \(U\_{k2}\) are coefficients estimated from the data, and \(w\) defines the number of cycles per unit time. In our study, \(w\) was fixed at 1/24 because one hour represents 1/24th of an animal’s daily activity cycle. Finally, the mixture weights for the first harmonic process, \(\alpha 1\), were modelled as the outcome of an exponential decay process, \(e^{-bt}\), where parameter \(b\) defines the decay rate of the exponential function. Thus, the proportional contribution of the post-collaring harmonic process declines from \(1\) at the time of collaring (\(t = 0\)) towards an asymptote of \(0\), which corresponds to a complementary increase in the importance of the baseline process.

This model is formulated as:

\[y\_t \sim Binomial(p\_t, N\_t)\] \[logit(p\_t) = \sum\_{k=1}^{2}\alpha\_{kt}(\mu\_k + U\_{k1}cos(2\pi wt) + U\_{k2}sin(2\pi wt))\]

\[\alpha\_{1t} = e^{-bt} ~~~ \alpha\_{2t} = 1-\alpha\_{1t}\] We assigned diffuse \(Uniform(0,5)\) and \(Uniform(-10,10)\) priors to represent post-collaring increases (\(a\)) and baseline logit-scale headshaking probability (\(c\)), respectively. The exponential decay rate, \(b\), was assigned a diffuse \(Uniform(-1,1)\) prior. As with other models, we calculated the halflife (\(log(2/b)\)) of the treatment effect, which represents the time required for the treatment effect to decline to half its initial magnitude.

We save our model parameterization as `3_NegExp_Harmonic_Binomial.R` to be loaded into **JAGS**.

```
model{
  
  # Priors
  b ~ dunif(-1,1)        # Exponential Decay Rate
  pi <- 3.14159265359    # Define pi
  omega <- 1/24          # Number of cycles/unit time
  mu[1] ~ dunif(-10,10)  # Baseline for Group 1 (the sequence with less headshaking)
  diff ~ dunif(-5,5)      # Specifying prior on the difference between group one and two (always positive)
  mu[2] <- mu[1] + diff  # Mean headshaking (on logit-scale) for group two is mean of group 1 plus 'diff'

  for (k in 1:2){  # Loop over groups (stressed/non-)
    U1[k] ~ dunif(-5,5)   # Harmonic coefficient - cos term
    U2[k] ~ dunif(-5,5)   # Harmonic coefficient - sin term
  }
  
  for (i in 1:nobs){
    y[i] ~ dbinom(p[i], K[i])
    logit(p[i]) <- w1[i]*(mu[1]+U1[1]*cos(2*pi*time[i]*omega) + U2[1]*sin(2*pi*time[i]*omega)) +
      (1-w1[i])*(mu[2]+U1[2]*cos(2*pi*time[i]*omega) + U2[2]*sin(2*pi*time[i]*omega))
    w1[i] <- exp(-b*time[i])
  } # i

  # Derived Quantities
  halflife <- log(2/b)
}
```

#### Model 3: Fit

We create a data list, define the parameters we want reported from the model, run the model in **JAGS**, and summarize results.

```
# Create Data List
data <- list(
  nobs = nrow(dat),
  y = dat$HDSHK,
  time = dat$X,
  K = dat$K,
  hour = dat$Hour
)

# Initial values
inits <- function(){
  list(
    b = 0.01,
    diff = -1,
    mu = c(-2,NA),
    U1 = c(1,1),
    U2 = c(1,1)
  )
}

params <- c('b','diff','mu','U1','U2','halflife','p')

# Execute jags model
mod2 <- jags(data = data, inits = inits, parameters.to.save = params, codaOnly = c("p"), model.file = "3_NegExp_Harmonic_Binomial.R", n.chains = 3, n.iter = n.iter, n.burnin = n.burnin, n.thin = n.thin, parallel=T)
```

```
## 
## Processing function input....... 
## 
## Done. 
##  
## Beginning parallel processing using 3 cores. Console output will be suppressed.
## 
## Parallel processing completed.
## 
## Calculating statistics....... 
## 
## Done.
```

```
print(mod2,3)
```

```
## JAGS output for model '3_NegExp_Harmonic_Binomial.R', generated by jagsUI.
## Estimates based on 3 chains of 2e+05 iterations,
## adaptation = 100 iterations (sufficient),
## burn-in = 40000 iterations and thin rate = 100,
## yielding 4800 total samples from the joint posterior. 
## MCMC ran in parallel for 1.379 minutes at time 2019-12-18 20:33:28.
## 
##              mean    sd     2.5%      50%    97.5% overlap0 f  Rhat n.eff
## b           0.124 0.007    0.110    0.124    0.140    FALSE 1 1.000  4800
## diff        3.046 0.068    2.900    3.050    3.166    FALSE 1 1.000  3670
## mu[1]      -8.623 0.052   -8.706   -8.630   -8.498    FALSE 1 1.001  1363
## mu[2]      -5.577 0.045   -5.665   -5.577   -5.489    FALSE 1 1.000  4800
## U1[1]       4.959 0.039    4.854    4.970    4.999    FALSE 1 1.001  3303
## U1[2]       1.419 0.051    1.318    1.420    1.519    FALSE 1 1.001  3960
## U2[1]       3.011 0.080    2.851    3.011    3.167    FALSE 1 1.001  1920
## U2[2]       0.220 0.043    0.134    0.221    0.300    FALSE 1 1.000  4800
## halflife    2.780 0.059    2.662    2.779    2.896    FALSE 1 1.000  4800
## deviance 2594.878 3.937 2589.097 2594.233 2604.311    FALSE 1 1.000  4800
## 
## Successful convergence based on Rhat values (all < 1.1). 
## Rhat is the potential scale reduction factor (at convergence, Rhat=1). 
## For each parameter, n.eff is a crude measure of effective sample size. 
## 
## overlap0 checks if 0 falls in the parameter's 95% credible interval.
## f is the proportion of the posterior with the same sign as the mean;
## i.e., our confidence that the parameter is positive or negative.
## 
## DIC info: (pD = var(deviance)/2) 
## pD = 7.8 and DIC = 2602.632 
## DIC is an estimate of expected predictive error (lower is better).
```

#### Model 3: Summarize and Plot

Calculate the highest posterior density intervals and create trace plots

```
# Look at trace and density plots to assess model convergence
MCMCtrace(mod2, ind=TRUE, params = c('b','diff','mu','U1','U2','halflife'), pdf=FALSE)
```

```
# Summarize results.  Include median
Post.Summary3 <- MCMCsummary(mod2, 
                             params = c('b','diff','mu','U1','U2','halflife'),
                             Rhat = TRUE,
                             n.eff = TRUE,
                             func = function(x) c(median(x),hdi(x,credMass = 0.95)),func_name = c('median','hdi_low','hdi_high'))

# View result summary
#Post.Summary3

# Plot results, including results from model 1 and model 2
#png(paste0(Output.dir,"Plot_",animals[id],".png"), width = 1000, height = 700, res=100)
par(mfrow=c(1,1))
plot(dat$X,dat$HDSHK/dat$K,type="b",xlim=c(0,nrow(dat)),pch = 15,cex = 0.5, las = 1, xlab="Hours since collaring", ylab="Headshaking", bty="n")

# Model 1
lines(dat$X,plogis(mod1$mean$a * exp(-mod1$mean$b*dat$X) + mod1$mean$c),type="l",col="red",lty=3,lwd=1)

# Model 2
lines(dat$X,(plogis(mod1.1$mean$a * exp(-mod1.1$mean$b*dat$X) + mod1.1$mean$c)*!(dat$Hour %in% 8:16)) + ((plogis(mod1.1$mean$a * exp(-mod1.1$mean$b*dat$X) + mod1.1$mean$c)*(dat$Hour %in% 8:16))^mod1.1$mean$d), type="l",lty=2,cex=1.2, lwd=1, col = "blue")

# Model 3
lines(dat$X,plogis(
  exp(-mod2$mean$b*dat$X)*(mod2$mean$mu[1] + mod2$mean$U1[1]*cos(2*pi*dat$X*1/24) + mod2$mean$U2[1]*sin(2*pi*dat$X*1/24)) +
    (1-exp(-mod2$mean$b*dat$X))*(mod2$mean$mu[2] + mod2$mean$U1[2]*cos(2*pi*dat$X*1/24) + mod2$mean$U2[2]*sin(2*pi*dat$X*1/24))),
  lty=1,lwd=2, col="dark green")

# Legend
legend(x = Llabel2[id],y = Llabel1[id], legend=c("Binomial [NExp Decay]","Binomial [NExp + Day/Night]","Binomial [NExp + Harmonic]"),lty=c(5,2,1), lwd=c(1,1,2), col = c("red","blue","dark green"), bty = "n")
```

```
#dev.off
```

#### Model 3: Calculate Metrics and Save

Calculate derived quantities from the results and save to the output directory

```
# Calculate:
# Background (recovery) rate for one 24-hour cycle
Recovered <- plogis(mod2$sims.list$mu[,2] + mod2$sims.list$U1[,2] %*% t(as.matrix(cos(2*pi*1/24*0:23))) + mod2$sims.list$U2[,2] %*% t(as.matrix(sin(2*pi*1/24*0:23))))
Recovered <- apply(Recovered, 1, mean)

# Headshaking rate immediately after collaring
Handled <- plogis(mod2$sims.list$mu[,1] + mod2$sims.list$U1[,1] %*% t(as.matrix(cos(2*pi*1/24*0:23))) + mod2$sims.list$U2[,1] %*% t(as.matrix(sin(2*pi*1/24*0:23))))
Handled <- apply(Handled, 1, mean)

# Treatment effect (percent increase)
Mod2EffectSize <- (Handled-Recovered)/Recovered*100

Recov.Stats <- Calc.Stats(Recovered)
Handle.Stats <- Calc.Stats(Handled)
Mod2EffectSize.Stats <- Calc.Stats(Mod2EffectSize)

# Bind the results to Post.Summary3 for export
Post.Summary3 <- rbind(Post.Summary3,Recov.Stats, Handle.Stats, Mod2EffectSize.Stats)

# Write file
write.csv(Post.Summary3, paste0(Output.dir,"/Model3_Halflife_Summary_",animals[id],".csv"))

# This is the end of the loop and must be activited iterate through each animal
#}
```

### GG-Plot

Results can also be summarized and plotted in ggplot2. This requires that you put all the data necessary into a dataframe, specifying the x and y axes.

```
# Input all details into a data.frame for GGPlotting
Output <- data.frame(x = dat$X,
                     y1 = plogis(mod1$mean$a * exp(-mod1$mean$b*dat$X) + mod1$mean$c),
                     y2 = (plogis(mod1.1$mean$a * exp(-mod1.1$mean$b*dat$X) + mod1.1$mean$c)*!(dat$Hour %in% 8:16)) + ((plogis(mod1.1$mean$a * exp(-mod1.1$mean$b*dat$X) + mod1.1$mean$c)*(dat$Hour %in% 8:16))^mod1.1$mean$d),
                     y3 = plogis(
                       exp(-mod2$mean$b*dat$X)*(mod2$mean$mu[1] + mod2$mean$U1[1]*cos(2*pi*dat$X*1/24) + mod2$mean$U2[1]*sin(2*pi*dat$X*1/24)) +
                         (1-exp(-mod2$mean$b*dat$X))*(mod2$mean$mu[2] + mod2$mean$U1[2]*cos(2*pi*dat$X*1/24) + mod2$mean$U2[2]*sin(2*pi*dat$X*1/24)))
)

# Create GGPlot
Plot.Result <- ggplot(dat, aes(x = X, y= HDSHK/K)) +
  #geom_point(aes(x = X, y= HDSHK/K)) + 
  geom_point(size = 0.5) + 
  #ylim(0,0.15) + 
  #xlim(0,100) + 
  ylab("Headshaking") + 
  xlab("Hours since collaring") + 
  geom_line(data = Output, aes(x = x, y = y1), lwd = 0.5, lty = 3, col="red") + 
  geom_line(data = Output, aes(x = x, y = y2), lwd = 0.5, lty = 2, col="blue") + 
  geom_line(data = Output, aes(x = x, y = y3), lwd = 0.75, col="dark green") + 
  theme_classic() + 
  theme(axis.title.y =element_text(family = "serif", size = 12),
        axis.title.x =element_text(family = "serif", size = 12),
        axis.text.y =element_text(family = "serif"),
        axis.text.x =element_text(family = "serif"),
        plot.title = element_text(hjust = -0.05, size = 12, family = "serif"))

# Plot results
Plot.Result
```

### Leave-One-Out Cross-Validation

To evaluate the fit of each model, we performed leave-one-out cross-validation. As with the example provided with our fecal glucocorticoid metabolite analysis (FGM), we must iterate through the entire dataset and remove a single record at each step. Models are then iteratively fit to each data subset, using the withheld record to validate individual models. We used thte sum of squared errors from Leave-One-Out Cross validation to compare fit across our three models.

```
# Setup cross-validation matrix to hold Sum of Squared Error Results
SSE <- matrix(nrow=4,ncol=3) # 4 animals, 3 models
rownames(SSE) <- unique(dat_all$Animal) # Input Animal Names
colnames(SSE) <- c("NegExp","DayNight","Harmonic") # Input models

# Loop over every individual
for (id in 1:4){
  # Subsets individual
  dat <- subset(dat_all, Animal==animals[id])
  # Further subsets the records before the secondary handling event. Don't want to model this second event
  dat <- subset(dat, X<=NextHandle[id])
  
  # Create matrix to hold the residual error
  errors <- matrix(nrow=dim(dat)[1], ncol=3)

  # Internal loop to separate data into training a testing.  The 'nrow()' of the dataset determines the number of models that will be fit (i.e., the length of the dataset).
  for (sample in 1:nrow(dat)){
    print(paste0(animals[id],": Sample ",sample," of ",nrow(dat)))
    
    train <- dat[-sample,]
    test <- dat[sample,]
    
    # ..... fit models and follow existing procedure above with the train dataset
 
    # Place residual errors in dataframe by comparing the test value with the modeled result 
    # Model 1: Negative Exponential Decay 
    errors[sample,1] <- test$HDSHK - test$K*plogis(mod1$mean$a * exp(-mod1$mean$b*test$X) + mod1$mean$c)
    
    # Model 2: Negative Exponential Decay with Day/Night Switch
    errors[sample,2] <- test$HDSHK - test$K*((plogis(mod1.1$mean$a * exp(-mod1.1$mean$b*test$X) + mod1.1$mean$c)*!(test$Hour %in% 8:16)) + ((plogis(mod1.1$mean$a * exp(-mod1.1$mean$b*test$X) + mod1.1$mean$c)*(test$Hour %in% 8:16))^mod1.1$mean$d)) 
    
    # Model 3: Negative Exponential Decay with Harmonics
    errors[sample,3] <- test$HDSHK - test$K*plogis(exp(-mod2$mean$b*test$X)*(mod2$mean$mu[1] + mod2$mean$U1[1]*cos(2*pi*test$X*1/24) + mod2$mean$U2[1]*sin(2*pi*test$X*1/24)) + (1-exp(-mod2$mean$b*test$X))*(mod2$mean$mu[2] + mod2$mean$U1[2]*cos(2*pi*test$X*1/24) + mod2$mean$U2[2]*sin(2*pi*test$X*1/24))
)
    # Export the file of errors
    write.csv(errors, paste0(Output.dir,"/Errors_id",id,".csv"))
    }
    # Sum the Squared Error of each column and place in matrix
    SSE[id,1:3] <- colSums(errors^2)
}
# Look at Results
SSE
```
