# Supplementary material for: Short-term effects of GPS collars on the activity, behavior, and adrenal response of scimitar-horned oryx (Oryx dammah)
Source: PLoS One. 2020 Feb 11;15(2):e0221843. doi: 10.1371/journal.pone.0221843 (PMC7012457; doi:10.1371/journal.pone.0221843)
Supplement: S1 Table — Parameter estimates from a multinomial regression model predicting animal behavior before (Pre-Treatment), during (Treatment), and after (Post-Treatment) periods for scimitar-horned oryx (n = 10) fit with GPS collars. Posterior mean, median, standard deviation (SD), and 95% credible interval (CI) provided. See Table 2 for behavior category descriptions. (DOCX) [file pone.0221843.s004.docx]

**S1 Table**: Parameter estimates from a multinomial regression model predicting animal behavior before (Pre-Treatment), during (Treatment), and after (Post-Treatment) periods for scimitar-horned oryx (n=10) fit with GPS collars. Posterior mean, median, standard deviation (SD), and 95% credible interval (CI) provided. See Table 2 for behavior category descriptions.

|  |  | *μ* | SD | median | 2.5% CI | 97.5% CI |
| --- | --- | --- | --- | --- | --- | --- |
| Standing (Head Up) | |  |  |  |  |  |
|  | Pre-Treatment | 0.47 | 0.05 | 0.47 | 0.35 | 0.55 |
|  | Treatment | 0.41 | 0.06 | 0.42 | 0.27 | 0.51 |
|  | Post-Treatment | 0.46 | 0.06 | 0.46 | 0.32 | 0.55 |
| Standing (Head Down) | |  |  |  |  |  |
|  | Pre-Treatment | 0.22 | 0.09 | 0.20 | 0.09 | 0.43 |
|  | Treatment | 0.27 | 0.10 | 0.25 | 0.12 | 0.50 |
|  | Post-Treatment | 0.28 | 0.10 | 0.26 | 0.12 | 0.51 |
| Laying | |  |  |  |  |  |
|  | Pre-Treatment | 0.02 | 0.02 | 0.01 | 0.00 | 0.06 |
|  | Treatment | 0.04 | 0.04 | 0.03 | 0.00 | 0.12 |
|  | Post-Treatment | 0.00 | 0.00 | 0.00 | 0.00 | 0.01 |
| Headshaking | |  |  |  |  |  |
|  | Pre-Treatment | 0.05 | 0.01 | 0.05 | 0.04 | 0.07 |
|  | Treatment | 0.08 | 0.01 | 0.08 | 0.05 | 0.10 |
|  | Post-Treatment | 0.03 | 0.00 | 0.03 | 0.02 | 0.04 |
| Locomotion | |  |  |  |  |  |
|  | Pre-Treatment | 0.14 | 0.03 | 0.14 | 0.08 | 0.21 |
|  | Treatment | 0.17 | 0.04 | 0.17 | 0.08 | 0.25 |
|  | Post-Treatment | 0.20 | 0.05 | 0.20 | 0.11 | 0.29 |
| Scratching | |  |  |  |  |  |
|  | Pre-Treatment | 0.10 | 0.02 | 0.10 | 0.06 | 0.16 |
|  | Treatment | 0.04 | 0.01 | 0.04 | 0.02 | 0.07 |
|  | Post-Treatment | 0.04 | 0.01 | 0.04 | 0.02 | 0.06 |
