# Supplementary material for: Short-term effects of GPS collars on the activity, behavior, and adrenal response of scimitar-horned oryx (Oryx dammah)
Source: PLoS One. 2020 Feb 11;15(2):e0221843. doi: 10.1371/journal.pone.0221843 (PMC7012457; doi:10.1371/journal.pone.0221843)
Supplement: S2 Table — Classification accuracy, precision and recall of behaviors identified by a random forest model in the analysis of accelerometry data (8 Hz) recorded in Vectronic GPS collars fit on four (n = 4) scimitar-horned oryx (Oryx dammah). (DOCX) [file pone.0221843.s005.docx]

S2 Table: Classification accuracy, precision and recall of behaviors identified by a random forest model in the analysis of accelerometry data (8 Hz) recorded in Vectronic GPS collars fit on four (n = 4) scimitar-horned oryx (Oryx dammah).

|  | Classified Results | | | | |
| --- | --- | --- | --- | --- | --- |
|  | Feeding | Headshaking | Resting | Locomotion | Total |
| Feeding | **2391** | 2 | 33 | 26 | 2452 |
| Headshaking | 0 | **78** | 1 | 0 | 79 |
| Resting | 9 | 6 | **2360** | 6 | 2381 |
| Locomotion | 0 | 1 | 6 | **2368** | 2375 |
| Total | 2400 | 87 | 2400 | 2400 | **7287** |
|  |  |  |  |  |  |
| Performance metrics |  |  |  |  |  |
| Precision | 99.63 | 89.66 | 98.33 | 98.67 |  |
| Accuracy | 99.04 | 99.86 | 99.16 | 99.46 |  |
| Recall | 97.51 | 98.73 | 99.12 | 99.71 |  |
|  |  | Overall Classification Accuracy: | | | 98.76 |
