# Supplementary material for: Short-term effects of GPS collars on the activity, behavior, and adrenal response of scimitar-horned oryx (Oryx dammah)
Source: PLoS One. 2020 Feb 11;15(2):e0221843. doi: 10.1371/journal.pone.0221843 (PMC7012457; doi:10.1371/journal.pone.0221843)

**S1 Fig**: Pearson’s correlation coefficients of animal behaviors identified within observation windows for captive scimitar-horned oryx (*Oryx dammah*). Head Up (HU), Headshaking (HDSK), Locomotion (LOCO), and Scratching (SCRATCH) most often occurred in combination with one another. Head Down (HD) and Laying (LAY) infrequently occurred in combination with these behaviors.


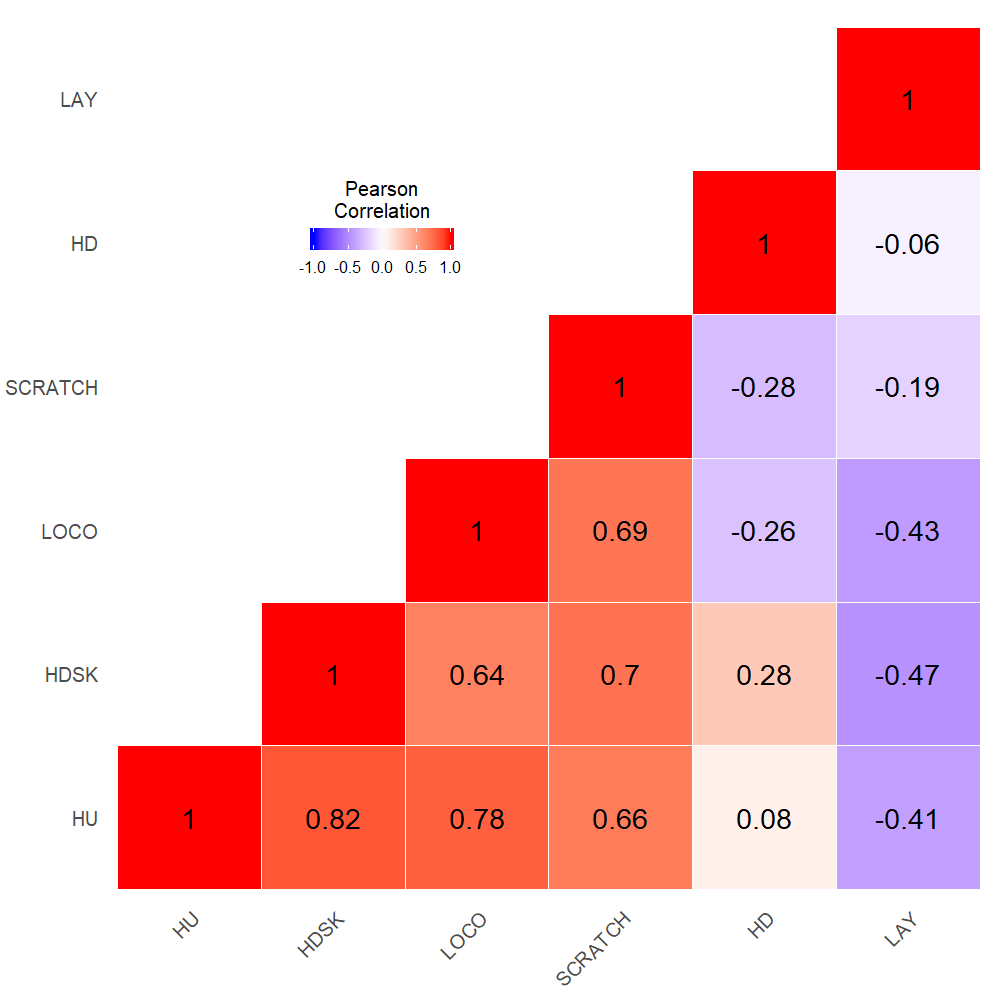

Supplement: S1 Fig — Pearson’s correlation coefficients of animal behaviors identified within observation windows for captive scimitar-horned oryx (Oryx dammah). Head Up (HU), Headshaking (HDSK), Locomotion (LOCO), and Scratching (SCRATCH) most often occurred in combination with one another. Head Down (HD) and Laying (LAY) infrequently occurred in combination with these behaviors. (DOCX) [file pone.0221843.s006.docx]
