# Supplementary material for: Short-term effects of GPS collars on the activity, behavior, and adrenal response of scimitar-horned oryx (Oryx dammah)
Source: PLoS One. 2020 Feb 11;15(2):e0221843. doi: 10.1371/journal.pone.0221843 (PMC7012457; doi:10.1371/journal.pone.0221843)

S1 Cross Validation: Leave-one-out cross-validation results from each of four (4) piecewise regression models, evaluating fecal glucocorticoid metabolites (n_obs_ = 55) collected from scimitar-horned oryx (*Oryx dammah*) fit with GPS collars (Treatment). Models fit in a Bayesian framework and evaluated by summing the squared errors (SSE). The Handling Response model was identified as the best model. That is, the Handling Response model minimized the Sum of Squared Error (noted in bold). We ran three parallel Markov chain Monte Carlo (MCMC) chains for 400,000 iterations, discarding the first 80,000 iterations (20%) of each chain as burn-in, and thinned the remaining posterior samples (1:100) from the joint posterior distribution for each model. See manuscript for further details.

Sum of Squared Errors:

Reducing the length of each MCMC chain (iterations: 20,000; burn-in: 4,000; thinning rate: 1:10), but fitting multiple repetitions of each model, resulted in the same conclusion – the Handling Response model minimized the Sum of Squared Errors. These leave-one-out cross-validation results emphasize variation within individual models, but highlight the robustness of conclusions, even when chains are shortened considerably.

Sum of Squared Errors:

Estimated parameters and predicted responses from each piecewise regression model (A - No Response; B - Stress Response; C - Habituation Response; D - Handling Response). We fit three parallel Markov chain Monte Carlo (MCMC) chains for 400,000 iterations for each model, discarding the first 80,000 iterations of each chain as burn-in. The remaining posterior samples were thinned at a rate of 1:100, yielding a total of 9,600 samples from the joint posterior distribution. Convergence was assessed by visual inspection of traceplots to ensure a reasonable exploration of the parameter space and by ensuring that the potential scale reduction factor was < 1.1 for each variable. See manuscript for details on each model.

1. No Response

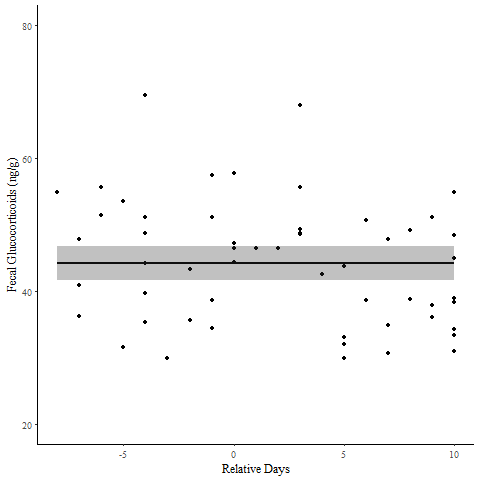


1. Stress Response

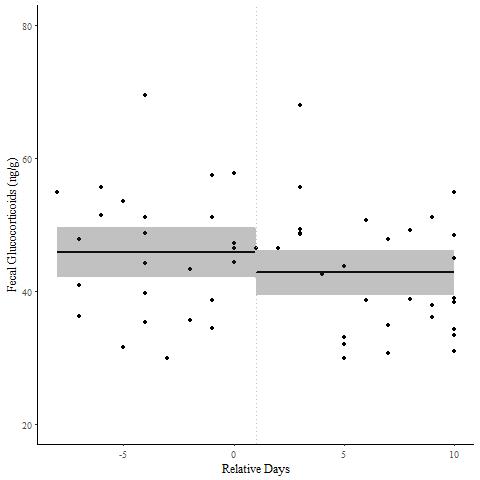


1. Habituation Response

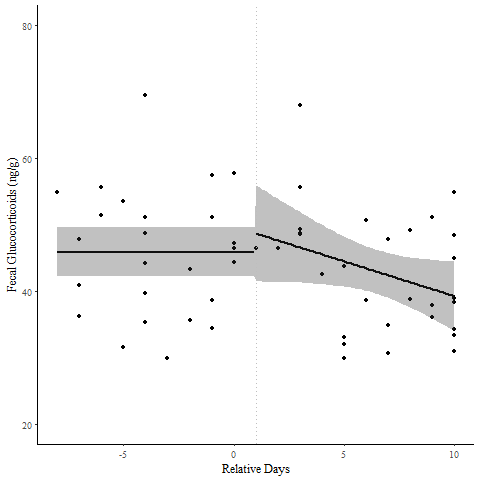


1. Handling Response (Top Model)

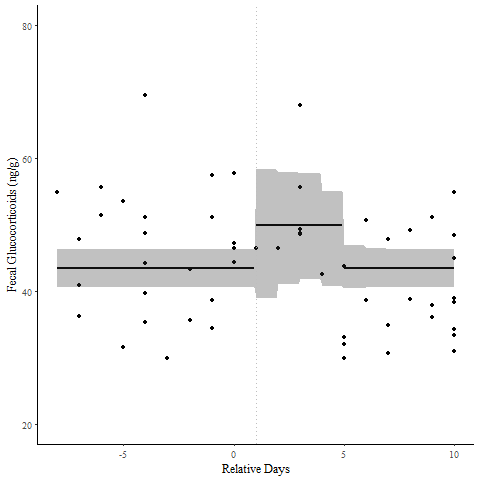

Supplement: S1 Cross Validation — Leave-one-out cross-validation results from each of four (4) piecewise regression models, evaluating fecal glucocorticoid metabolites (nobs = 55) collected from scimitar-horned oryx (Oryx dammah) fit with GPS collars (Treatment). Models fit in a Bayesian framework and evaluated by summing the squared errors (SSE). The Handling Response model was identified as the best model. We ran three parallel Markov chain Monte Carlo (MCMC) chains for 400,000 iterations, discarding the first 80,000 iterations (20%) of each chain as burn-in, and thinned the remaining posterior samples (1:100) from the joint posterior distribution for each model. (DOCX) [file pone.0221843.s007.docx]
