# Supplementary material for: Short-term effects of GPS collars on the activity, behavior, and adrenal response of scimitar-horned oryx (Oryx dammah)
Source: PLoS One. 2020 Feb 11;15(2):e0221843. doi: 10.1371/journal.pone.0221843 (PMC7012457; doi:10.1371/journal.pone.0221843)
Supplement: S3 Cross Validation — Leave-one-out cross-validation results evaluating three (3) time-series models fit to predict headshaking behavior in scimitar-horned oryx (Oryx dammah). Data derived from tri-axial accelerometers fit on four (n = 4) animals, recording eight activities per second (8 Hz). Headshaking was identified via random forest, an ensemble classification and regression tree. Results were aggregated to an hourly interval. Models predicting the number of hourly headshakes fit in a Bayesian framework and evaluated by summing the squared errors (SSE). The Harmonic model was identified as the best model (noted in bold) in three out of four cases. We ran three parallel Markov chain Monte Carlo (MCMC) chains for 400,000 iterations, discarding the first 80,000 iterations (20%) of each chain as burn-in, and thinned the remaining posterior samples (1:100) from the joint posterior distribution for each model. Predicted responses and estimated parameters from the joint posterior distributions are provided for each animal/model. (DOCX) [file pone.0221843.s009.docx]

S3 Cross Validation: Leave-one-out cross-validation results evaluating three (3) time-series models fit to predict headshaking behavior in scimitar-horned oryx (*Oryx dammah*). Data derived from tri-axial accelerometers fit on four (*n* = 4) animals, recording eight activities per second (8 Hz). Headshaking was identified via random forest, an ensemble classification and regression tree. Results were aggregated to an hourly interval. Models predicting the number of hourly headshakes fit in a Bayesian framework and evaluated by summing the squared errors (SSE). The Harmonic model was identified as the best model (noted in bold) in three out of four cases. We ran three parallel Markov chain Monte Carlo (MCMC) chains for 400,000 iterations, discarding the first 80,000 iterations (20%) of each chain as burn-in, and thinned the remaining posterior samples (1:100) from the joint posterior distribution for each model. Predicted responses and estimated parameters from the joint posterior distributions are provided for each animal/model. See manuscript for further details.

Sum of Squared Errors:

Estimable parameters and predicted responses from time-series regression models. Headshaking, determined by classifying raw data from tri-axial accelerometers and aggregated by hour, displayed in black (dots connected). Results separated between individuals (A – 114839; B - 114426; C – 114915; D - 114969). We fit three parallel Markov chain Monte Carlo (MCMC) chains for 400,000 iterations for each model, discarding the first 80,000 iterations of each chain as burn-in. The remaining posterior samples were thinned at a rate of 1:100, yielding a total of 9,600 samples from the joint posterior distribution. Convergence was assessed by visual inspection of traceplots to ensure a reasonable exploration of the parameter space and by ensuring that the potential scale reduction factor was < 1.1 for each variable. *Half-life* is the time (hours) required for the treatment effect (collar fitting) to decline to half its initial magnitude. *Treatment Effect* is the percent increase in headshaking when comparing the rate of headshaking after being fit with GPS collars (*Handling Rate*) with the background rate (*Recovery Rate*). That is, *Treatment Effect* = (*Handling Rate* – *Recovery Rate*)/*Recovery Rate* * 100. We assume the *Recovery Rate* to be equal to the pre-collaring (‘normal’) headshaking rate.

1. 114839

Predicted Responses


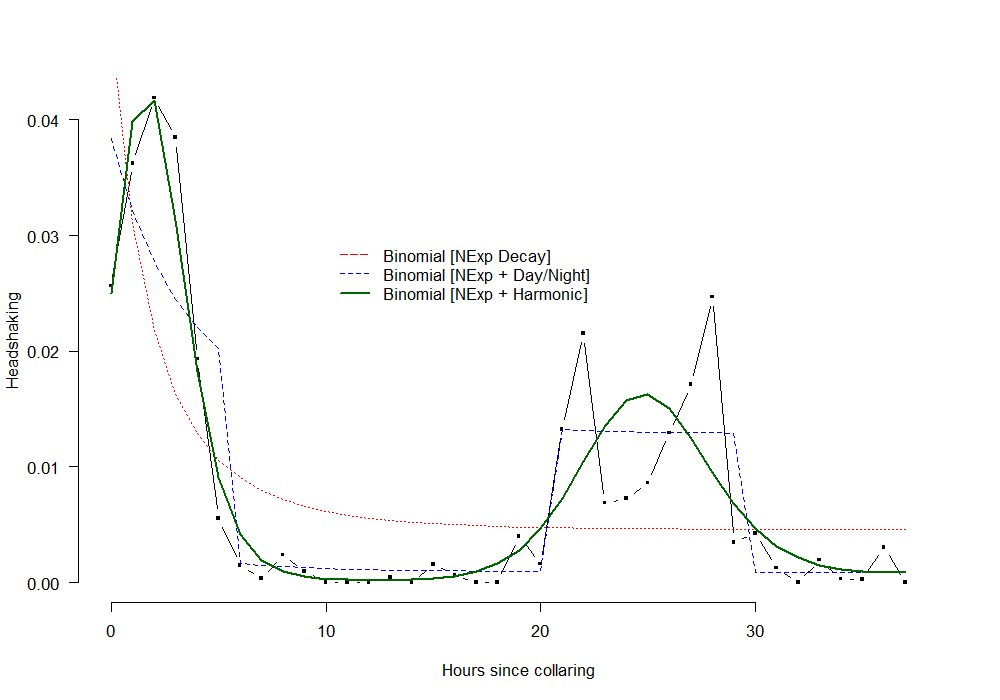


Estimated Parameters from Joint Posterior Distribution

1. 114426

Predicted Responses


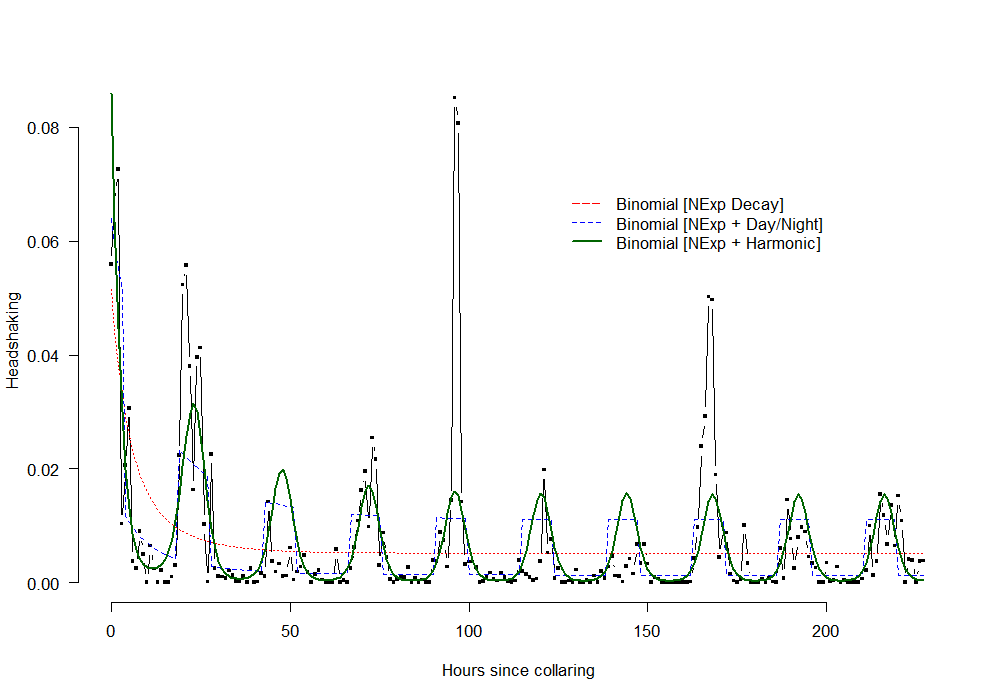


Estimated Parameters from Joint Posterior Distribution

1. 114915

Predicted Responses


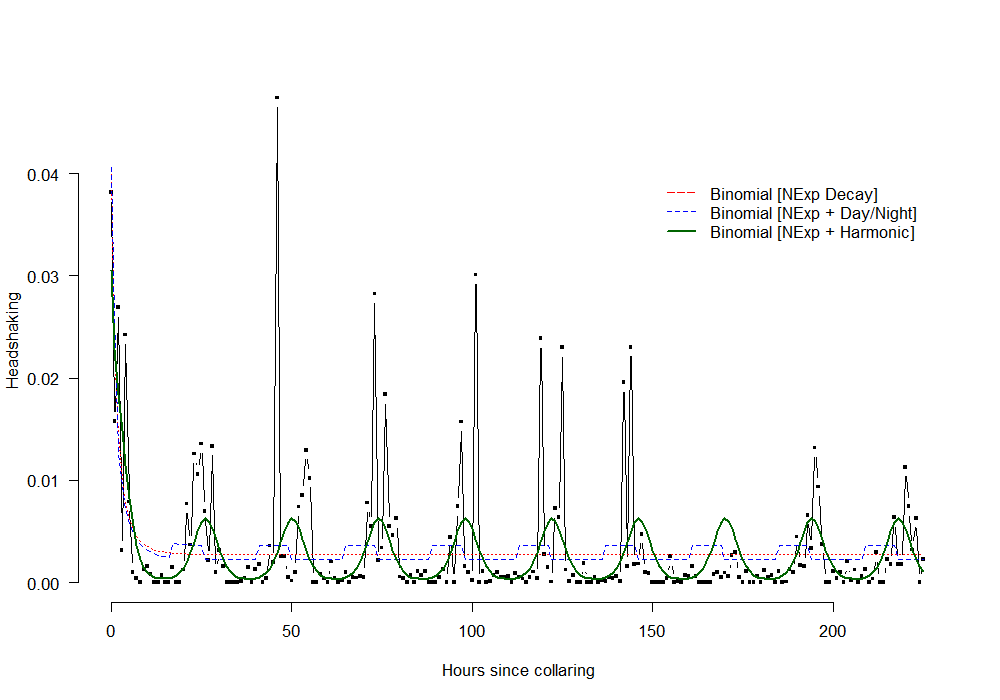


Estimated Parameters from Joint Posterior Distribution

1. 114969

Predicted Responses


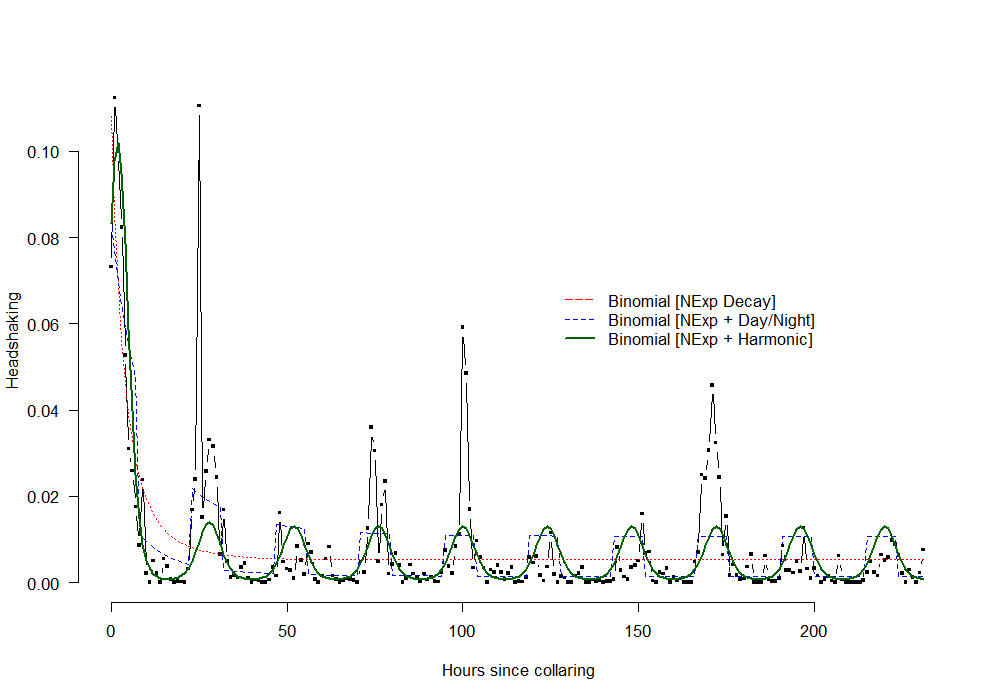


Estimated Parameters from Joint Posterior Distribution
